# Examining the use of evidence-based and social media supported tools in freely accessible physical activity intervention websites

**DOI:** 10.1186/s12966-014-0105-0

**Published:** 2014-08-17

**Authors:** Corneel Vandelanotte, Morwenna Kirwan, Amanda Rebar, Stephanie Alley, Camille Short, Luke Fallon, Gavin Buzza, Stephanie Schoeppe, Carol Maher, Mitch J Duncan

**Affiliations:** Central Queensland University, Centre for Physical Activity Studies, Rockhampton, QLD Australia; University of Western Sydney, School of Science and Health, Sydney, NSW Australia; University of South Australia, School of Health Sciences, Health and Use of Time Group, Adelaide, South Australia Australia

**Keywords:** Physical activity, Web-based intervention, Freely accessible, Web 2.0, Social media, Behavior change, Interactivity, User generated content, Website quality, Online, Internet

## Abstract

**Background:**

It has been shown that physical activity is more likely to increase if web-based interventions apply evidence-based components (e.g. self-monitoring) and incorporate interactive social media applications (e.g. social networking), but it is unclear to what extent these are being utilized in the publicly available web-based physical activity interventions. The purpose of this study was to evaluate whether freely accessible websites delivering physical activity interventions use evidence-based behavior change techniques and provide social media applications.

**Methods:**

In 2013, a systematic search strategy examined 750 websites. Data was extracted on a wide range of variables (e.g. self-monitoring, goal setting, and social media applications). To evaluate website quality a new tool, comprising three sub-scores (Behavioral Components, Interactivity and User Generated Content), was developed to assess implementation of behavior change techniques and social media applications. An overall website quality scored was obtained by summing the three sub-scores.

**Results:**

Forty-six publicly available websites were included in the study. The use of self-monitoring (54.3%), goal setting (41.3%) and provision of feedback (46%) was relatively low given the amount of evidence supporting these features. Whereas the presence of features allowing users to generate content (73.9%), and social media components (Facebook (65.2%), Twitter (47.8%), YouTube (48.7%), smartphone applications (34.8%)) was relatively high considering their innovative and untested nature. Nearly all websites applied some behavioral and social media applications. The average Behavioral Components score was 3.45 (±2.53) out of 10. The average Interactivity score was 3.57 (±2.16) out of 10. The average User Generated Content Score was 4.02 (±2.77) out of 10. The average overall website quality score was 11.04 (±6.92) out of 30. Four websites (8.7%) were classified as high quality, 12 websites (26.1%) were classified as moderate quality, and 30 websites (65.2%) were classified as low quality.

**Conclusions:**

Despite large developments in Internet technology and growth in the knowledge of how to develop more effective web-based interventions, overall website quality was low and the majority of freely available physical activity websites lack the components associated with behavior change. However, the results show that website quality can be improved by taking a number of simple steps, and the presence of social media applications in most websites is encouraging.

**Electronic supplementary material:**

The online version of this article (doi:10.1186/s12966-014-0105-0) contains supplementary material, which is available to authorized users.

## Introduction

Population physical activity levels remain low and are a major public health concern in high-income countries. The burden and cost of inactivity to society are high due to being associated with non-communicable chronic diseases such as cardiovascular disease, some cancers, diabetes, osteoporosis, and obesity [1,2]. Given the magnitude of the problem, effective large-reach low-cost physical activity interventions are needed [1]. Implementing interventions through the Internet has substantial potential, as Internet access in most high-income countries is very high. For example, 79% of Australians have access to broadband Internet [3], and it is estimated that in 2015 there will be over 2.8 billion Internet users worldwide [4]. There are many advantages of using the Internet for health promotion and preventive medicine, including options for instantaneous interactivity, continued assessment and follow-up, individual tailoring, diverse delivery formats (e.g. (printable-) text, video, audio, e-mail or combination), anonymity, and high convenience [5,6]. Moreover, Internet interventions can reach respondents nearly anywhere at any time through desktops, laptops and mobile devices [7,8].

However, achieving health behavior change through the Internet has proved to be considerably harder than anticipated [9]. While the potential of Internet technologies to change health behaviors has not yet been fully understood, web-based interventions still hold much promise. Recent reviews and meta-analyses in the field of physical activity show small and short-term effects in web-based interventions [10,11]. Long-term effects are lacking predominantly due to problems in attracting, engaging and retaining participants into web-based interventions [12,13]. However, much progress has been made and knowledge of what works and what doesn’t in web-based interventions has grown tremendously in recent years [13,14].

A number of strategies, frequently used in web-based behavior change interventions, are associated with positive health outcomes [13,15]. This includes strategies derived from behavior change theories such as the use of self-monitoring [16], goal setting [11,13], modeling [11], social support [14,16–18], and providing educational content [10]. But also the use of repeated contacts with participants [12,17], regularly updated websites [14], individually tailored feedback [11–13,17], e-mails [14,19], and alternative delivery modes (such as smartphone applications [11,20]) have shown to be effective components of web-based interventions. Furthermore, the level of ‘interactivity’ of behavior change websites itself has also shown to be essential for the effectiveness of web-based interventions [13,21,22].

In this respect the development and implementation of social media applications, which can be defined as a group of online applications that allow for the creation and exchange of user-generated content [23], have vastly increased the interactivity of many websites in recent years. Several types of social media-based websites can be distinguished; examples are: collaborative projects (e.g., Wikipedia), blogs or microblogs (e.g., Wordpress and Twitter), content communities (e.g., YouTube), and social networking sites (e.g., Facebook) [23]. These tools are a part of what was, in 2004, termed Web 2.0: the utilisation of the World Wide Web as a platform where content is continuously modified by all users in a collaborative fashion [23]. Whilst the evidence regarding the effectiveness of social media applications for physical activity interventions is still emerging [24–28], there are several reasons illustrating why using social media applications for promoting behavior change might be important. Firstly, social media applications can reach very large audiences (e.g. Facebook has 1.1 billion users each month) and have high levels of user engagement and retention [29]. The highly dynamic and flexible nature of social media applications, with continuously changing content, is likely partly responsible for this high popularity and engagement [30]. Secondly, information can be delivered via existing contacts (‘friends’), and this ‘word-of-mouth’ influence is more powerful than traditional social marketing strategies [31,32]. Thirdly, social media applications are characterized by user generated content and multidirectional communication flows in which users participate as both creators and consumers of web content [33]. This active engagement and content generation is more influential compared to websites that are passive or reactive in nature and which don’t allow users to generate new content [33]. Finally, social media applications can link people to physical activity opportunities, highlight previously unknown preferences among individuals, enable role-modeling, allow normative comparisons, and provide instantaneous feedback and reinforcement [24].

In 2003 Doshi et al. [15] conducted an evaluation of freely available physical activity promotion websites and concluded that the use of theory-based strategies was low, and that websites provided little assessment, feedback or individually tailored assistance. Also in 2003, Evers et al. [6] assessed existing health behavior change websites and concluded that many did not include the basic requirements to achieve health behavior change. When Doshi et al. [15] and Evers et al. [6] conducted their analyses, the field of web-based interventions was relatively undeveloped compared to the current understanding. Over the subsequent years the knowledge of what website features and components are likely to change behavior and increase user engagement has been guided by a wealth of original studies, systematic reviews and meta-analyses [11,12,14]. Given the continued proliferation of un-evaluated physical activity promotion websites, the increased knowledge of effective web-based intervention components, and the large developments in Internet technology, it is timely to re-assess to what degree current physical activity promotion websites incorporate evidence-based behavior change techniques and innovative social media features. This will provide insight into the current implementation of useful intervention components and will inform future work surrounding the dissemination of effective web-based interventions.

The aim of this study was to evaluate to what extent freely available physical activity promotion websites apply techniques that have shown to be conducive for behavior change in intervention studies and to evaluate, for the first time, to what extent they incorporate social media applications which allow for enhanced user interactivity.

## Methods

### Search strategy

A search strategy was developed to identify freely available physical activity promotion websites on the Internet. Firstly, a number of relevant search terms were identified through discussion among the research team and through trial and error with popular search engines. Only the most relevant search terms, in relation to the results they generated, were retained for use (see Table 1). Secondly, a search engine was determined. Three highly used search engines (Google, Bing and Yahoo) were compared in relation of the results they returned to the selected search terms. As there was a very large overlap in the results of these search engines, it was decided to only use the most popular search engine at the time (Google) to conduct the data collection. Thirdly, to further increase the robustness of the search strategy, and ensure as many as possible relevant physical activity promotion websites were identified, the ‘Google Trends’ application was used to identify the most related search-terms for each originally entered search term. Google Trends (www.google.com/trends) is a public website of Google Inc. that indicates how often a particular search-term is entered relative to the total search-volume across various regions of the world. Google Trends identified the top three related search-terms for each original search-term, and these were added to the search strategy (see Table 1). This resulted in a total of 25 search terms. Fourthly, only the first 30 results for each search term were included, as beyond this point the search results became increasingly unrelated and irrelevant. This resulted in 750 web addresses that were processed in the next stage of the study. The search was conducted on 15 March 2013.

**Table 1:** **Original and trend related search terms**

**Original search terms**	**Google trends related terms**
Exercise tracking	Fitness tracking
	Free exercise tracking
	Exercise software
Exercise tracker	Weight tracker
	Calorie exercise tracker
	Calorie tracker
Track my exercise	Nil.
Free exercise programs	Free weight programs
	Weight loss programs
	Online exercise program
Health tracking online	Nil.
Health tracking	Health tracking software
	Environmental health tracking
	Online health tracking
Free exercise websites	Nil.
Physical activity recording	Nil.
Exercise recording	Nil.
Walking tracker	gps walking tracker
	walking tracker app
	Walking distance tracker

### Website selection

**Figure 1:**
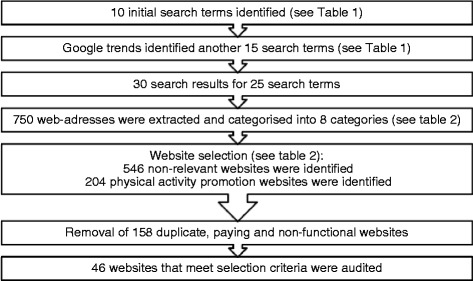
**Flow chart.**

**Figure 2:**
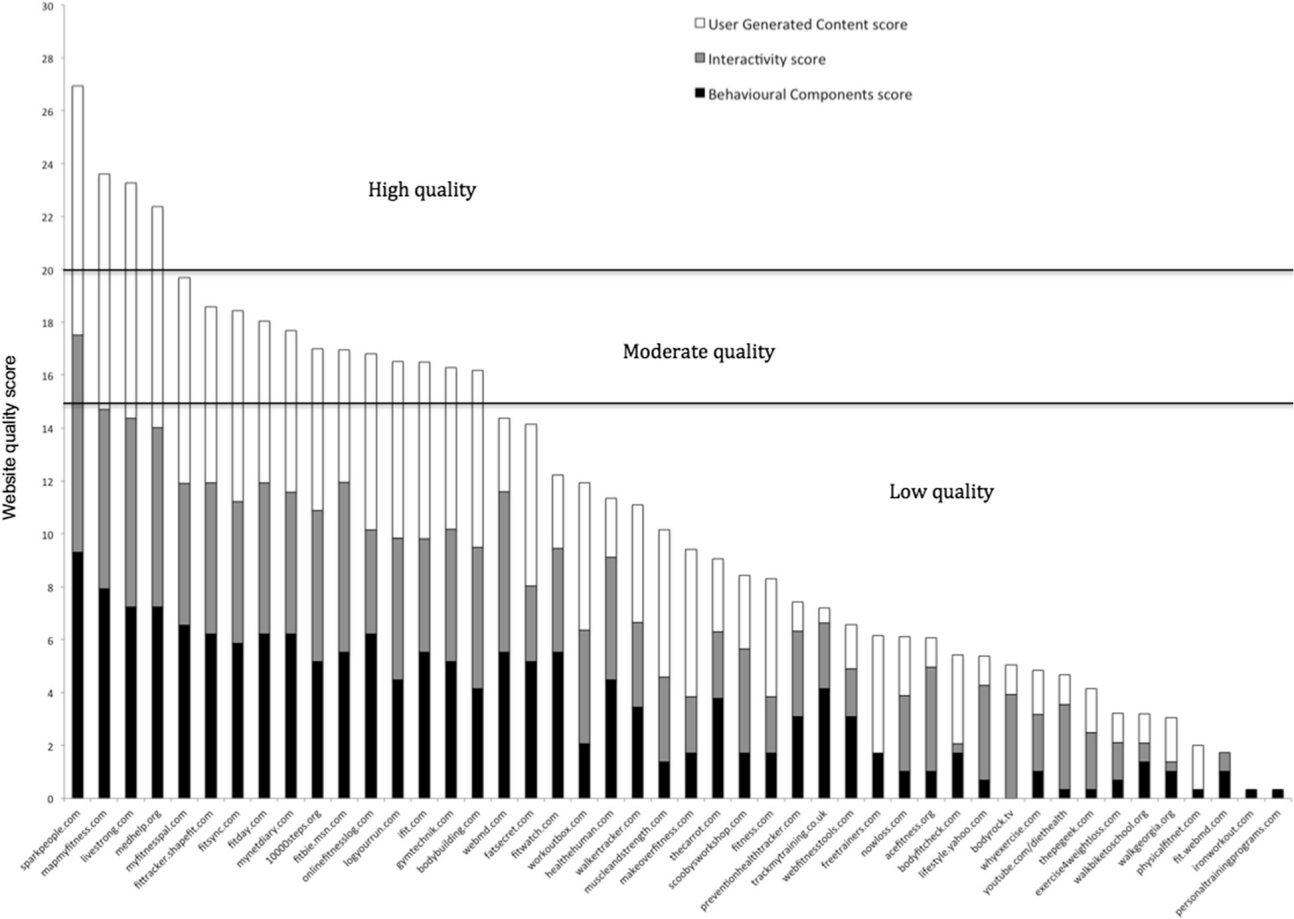
**Website quality according to the sub-scores of Behavioral Components, Interactivity and User Generated Content.**

The search results (n = 750, see Additional file 1 for all web-addresses) were exported to an Excel spreadsheet where they were categorized into eight categories of different types of website (see Table 2 for summary of categorization) by two members of the research team (LF, GB) who accessed all the web-addresses to determine the correct categorization. Only those websites categorized as ‘physical activity interventions’ were included in the final data extraction process. An overview of the website selection process is provided in Figure 1; the web-addresses of included websites are provided in Figure 2.

**Table 2:** **Website categories and definitions**

**Website type**	**Definition**	**Number identified**
1. Physical activity program	Web-based program that has the intention to help people to become more active and/or help people to live healthier, the goal being health behavior change	204
2. Link to activity program	Website which only provides a link to a web-based physical activity promotion program located on another website	104
3. Smartphone application	A website either directly or indirectly linked to an application used on a smartphone	116
4. Health information	A government or non-governmental website that only provides basic health information about physical activity	59
5. Article	Website that provides a media or scholarly article in relation to physical activity and health	60
6. Profile page	Website that provide profile pages of organisations (university and government) or people with organisations that refer to physical activity and health	25
7. Non physical activity program	Website that provides health program that is not related to physical activity	35
8. Other	For example, websites not related to health, websites providing commercial software, inactive websites (link does not work), commercial workplace health program, …	147
	**Total =**	**750**

### Data extraction

Data extraction concerned two main areas: (1) website features that have shown to be conducive for behavior change in interventions studies aiming to increase physical activity [34,35], and (2) interactive features that allow users to generate and share information and that can be grouped as social media applications [23,36]. Tables 3 and 4 provide an overview of all website features for which data was extracted.

**Table 3:** **Behavior change related website features**

**Website feature**	**Number (n = 46)**	**Percent**	**Sub-score**
Targeting other health behaviors (number):			
- Targeted only physical activity	5	10.9	
- Targeted one other behavior	6	13	
- Targeted two other behaviors	24	52.2	
- Targeted three or more other behaviors	11	24	
Targeting other health behaviors (what):			
- Weight loss	38	82.6	
- Diet or calories	37	80.4	
- Diabetes	10	21.7	
- High blood pressure	7	15.2	
- Cardio-vascular disease	5	10.9	
- Other diseases	16	36.8	
Tracking tools related to other behaviors:			
- Weight	26	56.5	
- Diet	19	41.3	
- Calories	17	37	
- Body mass index	16	34.8	
- Mood	6	13	
- Sleep	5	10.9	
- Stress	3	6.5	
- Tobacco use	1	2.2	
- Alcohol use, sitting time	0	0	
Educational information:			
- Provided general physical activity information	35	76.1	a
- Provided information on the benefits of physical activity	22	47.8	a
- Provided information on barriers to being active	3	6.5	a
- Provided references for the information provided	3	6.5	a
Physical activity tracking:			
- Provides any kind of physical activity assessment	27	58.7	a,b
- Provides a once off assessment, to assess current situation	26	56.5	a,b
- Provides ongoing self-monitoring of physical activity	25	54.3	a,b,c
- Allowed participants to set activity goals	19	41.3	a,b
Method used for physical activity tracking:			
- Online physical activity log	23	50	
- iPhone accelerometer	11	23.9	
- GPS	3	6.5	
- Heart rate monitor	1	2.2	
- Pedometer	1	2.2	
- Accelerometer (e.g. Fitbit, Jawbone UP)	0	0	
- Survey	0	0	
- Uses one method to track physical activity	12	26.1	
- Uses two methods to track physical activity	7	15.2	
- Uses three or more methods to track physical activity	4	8.8	
Type of physical activity tracking:			
- Time based (e.g. minutes)	23	50	a
- Activity based (e.g. walking)	22	47.8	a
- Intensity based (e.g. vigorous)	21	45.7	a
- Distance based (e.g. kilometres)	18	39.1	a
- Calorie based (e.g. Kcal)	13	28.9	a
- Other (e.g. steps, heart rate)	4	8.8	a
- One or two types of physical activity tracking	2	4.3	
- Three or four types of physical activity tracking	9	19.5	
- Five types of physical activity tracking	13	28.3	
Provision of feedback:			
- No form of feedback is being provided	25	54.3	
- Generic text-based feedback	23	50	a,b
- Generic image or graph based feedback	22	47.8	a,b
- Generic voice, video or avatar based feedback	0	0	a,b
- Targeted or tailored text-based feedback	1	2.2	a,b
- Targeted or tailored image or graph based feedback	2	4.3	a,b
- Targeted or tailored voice, video or avatar based feedback	0	0	a,b

Note. Sub-score: a = part of the Behavioral Components Score; b = part of the Interactivity Score; c = part of the User Generated Content Score.

**Table 4:** **Social media features**

**Website feature**	**Number (n = 46)**	**Percent**	**Sub-score**
Types of user generated content:			
- Sharing status updates	27	58.7	c
- Discussion forum	23	50	c
- Allowing personal user interactions	23	50	c
- Sharing success stories or testimonials	23	50	a,c
- Uploading photos or images	22	47.8	c
- Providing blogs	20	43.5	c
- Adding comments	18	39.1	c
- Share activity levels	10	21.7	c
- Invite new users to the site	9	19.6	a,c
- Google Map mash-ups	8	17.4	c
- No options for users to generate content	12	26.1	
- One or two options for users to generate content	9	19.6	
- Three to four options for users to generate content	4	8.7	
- Five to six options for users to generate content	6	13.1	
- Seven to eight options for users to generate content	9	19.6	
- Nine to ten options for users to generate content	6	13.1	
Gamification features:			
- Participation in challenges	9	19.6	b
- Creation of challenges	8	17.4	b,c
- Creation of activity teams	5	10.9	b,c
- Ability to earn points	4	8.7	a
- Leader board rankings	4	8.7	a
- Ability to earn trophies	3	6.5	a
- Ability to earn badges	2	4.3	a
- Ability to earn medals	2	4.3	a
- No gamification features available	35	76.1	
- One or two gamification features available	6	13.1	
- Three or more gamification features available	5	10.9	
Sharing of information with other websites through widgets:			
- No options to share through widgets	13	28.3	
- Facebook widget	32	69.6	b
- Twitter widget	27	58.7	b
- Pintrest widget	16	34.8	b
- Google widget	15	32.6	b
- e-mail widget	15	32.6	b
- Tumblr widget	7	15.2	b
- Linkedin widget	6	13.1	b
- Flicker widget	6	13.1	b
- Other (>5 for each): Research gate, Myspace, Blogger	22	47.8	
Has a presence on Facebook:			
- No Facebook presence	16	34.8	
- Low Facebook usage (<1 update/week)	3	6.5	b
- Medium Facebook usage (1 to 6 updates/week)	10	21.7	b
- High Facebook usage (≥7 updates/week)	17	37	b
Has a Twitter feed:			
- No Twitter feed	24	52.2	
- Low Twitter usage (<1 update/week)	3	6.5	b
- Medium Twitter usage (1 to 6 updates/week)	4	8.7	b
- High Twitter usage (≥7 updates/week)	15	32.6	b
Has a YouTube channel:			
- No YouTube channel	24	52.2	b
- Has between 0 and 10 videos on channel	7	15.2	
- Has between 11 and 30 videos on channel	4	8.7	
- Has more then 30 videos on channel	11	23.9	
Has a smartphone application			
- No smartphone application	30	65.2	
- Has iOS (Apple) application	16	34.8	b
- Has Android (Google) application	6	13.1	b
- Has a free smartphone app	11	23.9	
- Has a commercial smartphone app (range: $1.99- $4.99)	5	10.9	
Other social media features:			
- Has a ‘Wiki’	1	2.2	b,c
- Has an ‘RSS feed’	13	28.3	b
- Has ability to log in via social media integration	14	30.4	b

Note. Sub-score: a = part of the Behavioral Components Score; b = part of the Interactivity Score; c = part of the User Generated Content Score.

Data extraction was undertaken using purpose-designed data extraction forms which listed all the website features for which data needed to be extracted. The forms allowed coders to extract data from the selected websites in a convenient and consistent way. At the start of the data extraction, data for five websites was extracted independently by two members of the research team (LF, GB). This data was compared and the agreement between coders was very high (Cohen’s Kappa Inter-coder reliability (*k*) was 0.98 indicating nearly complete agreement). Data extraction for the remaining websites was undertaken by one researcher (either LF or GB), with coders consulting each other for all cases where appropriate coding was ambiguous. When the coders could not come to an agreement they consulted other members of the research team (MD, MK, CV), and a decision was made based on consensus.

### Quality assessment

Three sub-scores were developed to assess the overall quality of the websites that were assessed. The *Behavioral Components score* measured the presence of website components that have shown to be conducive for behavior change in interventions studies aiming to increase physical activity [34,35,37]. The *Interactivity score* measured the presence of website components that allowed users to interact with the website, as previous studies have shown that high levels of website interactivity are related to higher levels of behavior change [22]. The *User Generated Content score* measured the presence of website components that allowed users to generate new content on the website, either only accessible to a specific user (e.g. in a user profile), a user subgroup (e.g. ‘friends’) or all those that use the website. The ability for users to generate content is unique to social media applications [23], and represents a higher level of interactivity and potential for social support than websites that do not allow this.

Tables 3 and 4 indicate which website features were part of what sub-scores; when a feature was present on a website it scored ‘1’ , when it is absent it scored ‘0’. Some website features were part of more than one of the sub-scores (e.g. as self-monitoring has shown to contribute to behavior change, provides website interactivity and/or allows users to generate new content simultaneously it was part of all three sub-scores). Overall website quality was obtained by summing the three sub-scores. However, the Behavioral Component, the Interactivity and the User Generated Content scores are made up of a different number of items: 27, 33 and 14 items respectively (see Tables 3 and 4). Therefore, to ensure that each sub-score weighted equally in the overall website quality score they were recalculated so that each sub-score would only contribute 1/3 of the overall website quality score. To do this, each sub-score was divided by the total amount of items included in the sub-score and multiplied by 10. Thus, the minimum for each sub-score was 0 and the maximum score was 10 (e.g. for the Behavioral Components score: [(score obtained by website/27) * 10]). As such, when summing the three sub-scores, the overall website quality score ranged from 0 to 30. In relation to this, items that were part of more than one sub-score (see Tables 3 and 4 for which items this applies) thus weighted more heavily on the overall website quality score. When items are part of more than one sub-score it means they influence several factors simultaneously (e.g. behavior change and interactivity) and are therefore considered more important than items that are part of one sub-score only. Websites that obtained 66.7% or higher of the maximum score (being 30) were categorized as ‘high quality websites’; those obtaining a score between 50% and 66.6% of the maximum score were categorized ‘moderate quality websites’; and those obtaining a score lower than 50% of the maximum score were categorized as ‘low quality websites’ [38].

### Analyses

Basic descriptive statistics were used to report on the prevalence of the different website features across all categories.

## Results

Of the 750 websites that were extracted through the Google search engine, 204 were initially identified as physical activity promotion websites (see Figure 1). However, upon closer inspection 158 of those were discarded based on either being a duplicate, non-functional or a commercial website that required payment for access to the website. As such, 46 websites remained and were subjected to the data extraction method. The following sections only relate to this final sample of websites.

### Features related to behavior change

Few of the assessed websites (10.9%) focused solely on physical activity; more than half (52.2%) focused on two additional behaviors (see Table 3). In addition to physical activity, diet and weight loss were targeted in 80.4% and 82.6% of the websites respectively. Many websites also provided specific tools to self-monitor or track these additional behaviors, with the two most popular alternative tools being a weight tracker (available on 56.5% of all websites) and a dietary tracking tool (available on 41.3% of all websites).

The majority of assessed websites provided educational information about physical activity (76.1%), which included information on the benefits of being active in about half of the websites (47.8%). However, few websites provided information on how to overcome barriers to physical activity (6.5%) or provided information regarding where this educational information was sourced (6.5%).

Just over half of all websites (54.3%) provided tools for ongoing self-monitoring of physical activity, whereas only 41.3% of websites allowed participants to set activity goals. Among those websites that allowed ongoing self-monitoring, half (26.1% of all websites) provided only one option for participants to track their activity level, whereas the remaining websites (24% of all websites) provided two or more options. The most popular tracking tool was a physical activity log, available in nearly all websites that provided self-monitoring (50% of all websites), followed by self-monitoring using an inbuilt smartphone accelerometer (23.9% of all websites). Almost all websites that provided self-monitoring offered to track physical activity using multiple activity indicators, such as time-, distance-, intensity- and calorie-based tracking methods.

More than half of all websites (54%) did not provide any feedback to participants about their physical activity behavior, and of those that did the majority provided generic text-based (50%) or image/graph-based (47.8%) feedback, with only a very small number of websites offering targeted or tailored feedback (text-based: one website; image/graph-based: two websites). None of the websites offered feedback using voice (e.g. podcast), video or avatars.

### Social media applications

More than 70% of the websites offered options for users to generate content; 45.8% off all websites had five or more options for users to share content (see Table 4). The most popular options for content sharing were social media status updates (58.7%), discussion forums (50%), sharing success stories (50%), uploading photos (47.8%), blogs (43.5%) and the option to make comments (39.1%).

Gamification features (website components that have game like features) were less frequently used; less than one quarter of websites (23.9%) had some gamification features. Though most websites that did use gamification features had more than one such feature available for users. Most popular gamification features were in relation to the creating and participating in challenges as individuals or teams.

Most websites (71.7%) provided options to share content of their website with other websites through widgets, with Facebook (69.6%) and Twitter (58.7%) being the most popular websites content could be shared to. All the websites that embedded widgets provided more than one option for users to share content with external websites. Many websites also had their own Facebook page (65.2%) or a Twitter Feed (47.8%) and the majority of those were characterized by high usage (updates are provided at least once a day), as the page administrator provided frequent page updates or ‘tweeted’ regularly. Nearly a third of websites (30.4%) also allowed users to log into their website using their password and username from another website, such as Facebook; this social media integration makes it more convenient for people to use their website.

Approximately half of websites (47.8%) had a ‘YouTube’ channel, the majority of which had more than 30 videos available for users to watch. Just over one third of the websites (34.8%) had their own smartphone application; all of which had an application supported by Apple’s iOS platform, while only six websites offered an application supported by Google’s Android platform. Two thirds of the smartphone applications were offered for free to users, whereas the other third required a fee ranging from $1.99 to $4.99.

### Website quality

The average Behavioral Components score was 3.45 (±2.53) out of 10; four websites (8.7%) were classified as high (score was 6.67 or higher) on this sub-score, 13 websites (28.2%) were classified as moderate (score was between 5 and 6.66) and 29 websites (63.0%) were classified as low (score was lower than 5). The average Interactivity score was 3.57 (±2.16) out of 10; four websites (8.7%) were classified as high on this sub-score, 11 websites (23.9%) were classified as moderate and 31 websites (67.4%) were classified as low. The average User Generated Content score was 4.02 (±2.77) out of 10; 11 websites (23.9%) were classified as high on this sub-score, nine websites (19.5%) were classified as moderate and 26 websites (56.5%) were classified as low. The average overall website quality score was 11.04 (±6.92) out of 30 (see Figure 2); four websites (8.7%) were classified as high quality, 12 websites (26.1%) were classified as moderate quality, and 30 websites (65.2%) were classified as low quality.

## Discussion

This study evaluated freely available physical activity promotion websites to inform future work surrounding the dissemination of web-based interventions. The findings from this study may be useful for consumers, researchers, health care providers and website developers. The outcomes of this study illustrate that there are many free physical activity websites available on the Internet which use a wide variety of behavior change and social media components; however website quality was predominantly low aside from a small number of websites with good overall quality.

Only two of the 24 websites (www.ifit.com and www.webMD.com) reviewed by Doshi et al. [15] were among the 46 websites reviewed in this study and have continued to be available over the 10-year time gap between reviews. Although using a different assessment tool, the quality of one of these websites seemed to have improved over time (both were categorised as having low quality in the Doshi et al. [15] study; www.ifit.com was classified as having moderate quality in this study). The small overlap between both studies illustrates the dynamic and rapidly changing nature of the Internet. Despite this, many of the outcomes of Doshi et al. [15] are to a large extent comparable with the current study. Doshi et al. [15] also found that most websites provided sufficient educational information, but that the use of evidence-based strategies was low, and that websites provided little individually tailored assistance. Similarly, Doshi et al. [15] also found that a handful of websites out-performed the majority, and that there is substantial variation in physical activity websites with regard to behavior change offerings.

Similar studies in related fields found comparable outcomes demonstrating low website quality and capacity to change behavior. Evers et al. [6] who evaluated websites for a range of health behaviors reported low implementation of web-based components that provide tailored feedback, theory and interactivity. Bonar-Kidd et al. [39] who focussed on physical activity websites for cancer prevention indicated that only 4 (22%) of the 17 websites they surveyed were of high quality and concluded that information seekers are at risk for disease and injury. Shanar et al. [40] assessed websites focussing on diet and nutrition and indicated that 54% of websites had low quality and that there is great concern about the accuracy of the information being disseminated. Gorczynski et al., [41] who examined the quality of physical activity, exercise and sport information for people with schizophrenia, reported that most websites (59%) they examined did not provide information supported by any physical activity guidelines and that few websites (29%) discuss cognitive or behavioral aspects that could promote physical activity participation. This finding is in line with a study reporting that websites for breast cancer prevention lack behavior change components that impede on users’ motivations to protect themselves against breast cancer [42]. The present study builds on these previous reviews by also examining social media components as part of the overall assessment of website quality, and as it is the first study to do so it is not possible to compare outcomes with other studies on this specific dimension of website quality.

The majority (89.1%) of included websites addressed multiple health behaviors, and allowed tracking of these behaviors simultaneously; this was also observed in the study by Evers et al. [6] that reported that 78% of websites addressed multiple behaviors. There is some evidence indicating that interventions that address multiple health behaviors simultaneously are equally or more effective compared to those who address behaviors sequentially [43,44], as such it may be a positive observation to see so many websites addressing multiple health behaviors.

Despite the importance of established behavior change techniques (self-monitoring [17], goal setting [13], and providing feedback [11]), and the ability to easily implement them through the Internet, only about half of the included websites provided these features. Websites lacking such features are as such seriously limiting the potential for behavior change. The overall lack of targeted or tailored feedback was especially concerning, as there is considerable evidence supporting the effectiveness of personally relevant feedback [45]. While it is not difficult to provide targeted or tailored feedback from a technical point of view, developing targeted and tailored content in itself is complex and requires a deep understanding of the target behavior, as well as a good knowledge of health behavior change principles and theories. It may be that developers and providers in the field lack this expertise and therefore refrain from providing targeted and tailored feedback.

There is substantial evidence that web-based interventions are increasingly more effective if they incorporate more behavior change techniques [11,37]. Therefore it is positive to observe in Figure 2 that, despite the lack of basic behavior change tools in many websites, the majority of websites deployed a range of tactics to persuade users to become more active. As such, it was also encouraging to observe that nearly all websites incorporated features that allowed users to generate new content themselves. In fact, the User Generated Content sub-score was higher than the other sub-scores, and there were more websites with a high score (n = 11) on this scale when compared to the other scales. Webb et al. [11] reported that the effectiveness of web-based interventions is enhanced when additional methods of communicating with participants are used. Therefore the large and active presence of the included websites on platforms such as Facebook, Twitter and YouTube, as well as the use of social media integration and smartphone applications are positive trends that should be encouraged. Developers and health promoters should nevertheless be aware that user demographics of popular social media platforms are dynamic and continuously changing, as is the mode on how the Internet is accessed with a rapid increase in the use of mobile devices. Furthermore, the proliferation and change in web-applications is driven by a continuous interaction with user demand/experience/feedback and by technological (hard- and software) innovations. In order to be effective and reach intended audiences, web-based interventions will continuously need to adapt to these changes.

There are several study limitations. First, the Internet is a dynamic environment; conducting the web-search on another day may have resulted in different search outcomes. As such, the outcomes of this study provide only a momentary snapshot. Longitudinal reviews are needed to determine if websites are improving and how web-based physical activity promotion is changing over time. Second, the website quality outcomes obtained in this study are not linked to studies assessing effectiveness and maintenance of behavior change. A website with a higher quality score may not necessarily be more effective in increasing physical activity when compared to a website with a lower quality score. It may be that some websites included fewer website components, but implement them particularly well. Similarly, this study did not assess how quality was related to usage as some websites that scored poorly may have a large user-base. Further, the actual content of the websites was not assessed for correctness. In line with this, the videos provided by websites having a YouTube channel may have provided important behavioural or educational information over less relevant promotional information. As video content was not assessed, this is another limitation of the website quality scoring system. Third, this study only assessed free and publicly available websites. It may be that commercial websites with paying consumers have higher quality with regards to behavior change components and use of web social media applications. Finally, this study only examined English websites; website quality of non-English websites may differ. This study also has strengths. An innovative assessment tool that is in tune with how the Internet is being used today was developed. It assessed both new social media applications and established behavior change techniques. To our knowledge this is the first study to review the use of social media components in freely available physical activity websites. Further, the inter-coder reliability for included websites was very high, which provides confidence in the results presented.

## Conclusion

Ten years ago Evers et al. [6] concluded that the Internet was in the early phases of development with regards to health behavior change, as most programs readily available to consumers did not provide the basic components necessary for health behavior change. Despite large developments in Internet technology and large growth in the knowledge of how to design and implement web-based physical activity interventions [13,14], the majority of freely available physical activity websites still appear to be lacking the components and features likely to produce behavior change. However, the results of this study illustrate that website quality can be improved by taking a number of simple steps, and the presence of features that allow users to generate content in most websites is encouraging.

## Additional file

Additional file 1:
**Full list of web-addresses (n = 750) retrieved as a result of the search strategy.**
