# Supplementary material for: Examining the use of evidence-based and social media supported tools in freely accessible physical activity intervention websites
Source: Int J Behav Nutr Phys Act. 2014 Aug 17;11:105. doi: 10.1186/s12966-014-0105-0 (PMC4158049; doi:10.1186/s12966-014-0105-0)
Supplement: Additional file 1: — Full list of web-addresses (n = 750) retrieved as a result of the search strategy. [file 12966_2014_105_MOESM1_ESM.pdf]

Appendix 1: Full list of web-addresses (n = 750) retrieved as a result of the search stragety.

Search term	Page rank	Web-address	Website category
Exercise Tracking	1	http://lifehacker.com/5257812/six-best-exercise-planning-and-tracking-tools	Links to Online Health Program
	2	http://singularityhub.com/2012/10/15/the-fitbit-zip-a-60-exercise-tracking-clipon-that-automatically-syncs-to-your-devices/	Links to Online Health Program
	3	http://www.myfitnesspal.com/	Physical activity Online Health program
	4	http://www.lifehacker.com.au/2009/05/five-best-exercise-planning-and-tracking-tools/	Links to Online Health Program
	5	http://www.justwalk.com/	Physical activity Online Health program
	6	http://www.fitwatch.com/diary/activitydiary.html	Physical activity Online Health program
	7	http://www.skimble.com/	App
	8	http://www.gymheroapp.com/	App
	9	http://www.fitday.com/	Non PA Online Health Program
	10	http://www.appszoom.com/android_applications/exercise+tracking	App
	11	http://www.livestrong.com/article/383967-diet-and-exercise-tracking/	Physical activity Online Health program
	12	http://dailyburn.com/	Physical activity Online Health program
	13	https://itunes.apple.com/us/app/jefit-workout-fitness-bodybuilding/id449810000?mt=8	App
	14	http://www.onlinefitnesslog.com/	Physical activity Online Health program
	15	http://faboh.com/ets/	Business level online health program
	16	http://area.autodesk.com/flame-tutorials-legacy/exercise_tracking_camera_motion-4	Other
	17	http://support.loseit.com/customer/portal/questions/628929-exercise-tracking	NGO health information
	18	http://blog.visual.ly/visualizing-fitness-data/	Links to Online Health Program
	19	http://lifehacker.com/5907870/five-best-fitness-tracking-appliances	Links to Online Health Program
	20	http://www.startwalkingnow.org/mystart_tracker.jsp	Government health information
	21	http://physiqueapp.com/	App
	22	http://www.washingtonpost.com/blogs/the-checkup/post/usdas-new-diet-and-exercise-tracking-tool/2010/12/20/gIQArHI9IP_blog.html	Links to Online Health Program
	23	http://www.nytimes.com/2012/07/29/technology/nike-fuelband-tracks-physical-activity-inconsistently.html	Links to Online Health Program
	24	http://blog.withings.com/en/2012/10/04/mynetdiary-and-withings-partner-for-calorie-and-exercise-tracking/	Online Health program
	25	http://www.noom.com/tracking/	App
	26	http://allaboutwindowsphone.com/flow/item/15404_ProAktivo_provides_m	Links to Online Health Program

		obile_cent.php	
	27	http://www.hokiewellness.hr.vt.edu/PhysicalHealth/ExerciseFitness/ExerciseTracking.aspx	Links to Online Health Program
	28	http://dailytekk.com/2012/06/11/fitness-tracking-devices/	Links to Online Health Program
	29	https://itunes.apple.com/us/app/workout-log-fitness-exercise/id396267216?mt=8	App
	30	http://www.148apps.com/news/favorite-apps-track-exercise/	App

Exercise tracker	1	http://www.myfitnesspal.com/	Physical activity Online Health program
	2	http://lifehacker.com/5257812/six-best-exercise-planning-and-tracking-tools	Links to Online Health Program
	3	http://www.fitwatch.com/diary/activitydiary.html	Physical activity Online Health program
	4	http://www.mynetdiary.com/	Physical activity Online Health program
	5	http://www.fitday.com/	Non PA Online Health Program
	6	http://www.webmd.com/diet/food-fitness-planner/default.htm	Physical activity Online Health program
	7	https://itunes.apple.com/au/app/exercise-tracker/id293603711?mt=8	App
	8	http://download.cnet.com/MedHelp-s-Exercise-Tracker/3000-2056_4-10909577.html	Links to Online Health Program
	9	http://www.wired.com/playbook/2012/08/fitness-trackers/	Links to Online Health Program
	10	http://www.medhelp.org/land/exercise-tracker	Physical activity Online Health program
	11	http://www.healthehuman.com/features/exercise-workout-tracker	Physical activity Online Health program
	12	http://www.webfitnesstools.com/	Physical activity Online Health program
	13	http://www.livestrong.com/article/362134-diet-exercise-trackers/	Physical activity Online Health program
	14	http://appworld.blackberry.com/webstore/content/27743/?lang=en	App
	15	http://fittracker.shapefit.com/	Physical activity Online Health program
	16	http://www.gymheroapp.com/	App
	17	http://apps.facebook.com/fit-ify/	Other
	18	http://faboh.com/ets/	Business level online health program
	19	https://itunes.apple.com/us/app/fitocracy-free-daily-workout/id509253726?mt=8	App
	20	http://dailyburn.com/	Physical activity Online Health program
	21	http://www.startwalkingnow.org/	Government health information
	22	http://www.flawlesspt.com.au/index.php?option=com_content&view=article&id=217&Itemid=110	Physical activity Online Health program
	23	https://play.google.com/store/apps/details?id=com.gymrat&hl=en	App
	24	http://healthland.time.com/2012/12/04/top-10-health-lists/slide/workout-tracker/	Links to Online Health Program
	25	https://www.getfittn.com/	Government health information

	26	http://www.logyourrun.com/runninglog/	Physical activity Online Health program
	27	http://www.dairycouncilofca.org/Tools/TeenBeat/	Non PA Online Health Program
	28	http://www.skimble.com/	App
	29	https://itunes.apple.com/us/app/jefit-workout-fitness-bodybuilding/id449810000?mt=8	App
	30	http://blackberry-applications.toptenreviews.com/health-and-fitness/exercise-tracker-review.html	Links to Online Health Program

Track my exercise	1	http://www.mapmyfitness.com/	Physical activity Online Health program
	2	http://www.myfitnessjournal.com/	Physical activity Online Health program
	3	http://www.justwalk.com/	Physical activity Online Health program
	4	http://www.myfitnesspal.com/	Physical activity Online Health program
	5	http://lifehacker.com/5257812/six-best-exercise-planning-and-tracking-tools	Links to Online Health Program
	6	http://www.makeuseof.com/dir/fitnesstracker-track-daily-exercise/	Links to Online Health Program
	7	http://fit.webmd.com/kids/move/article/track-your-exercise	Physical activity Online Health program
	8	http://www.webmd.com/diet/food-fitness-planner/default.htm	Physical activity Online Health program
	9	https://www.exercise.com/article/why-you-should-track-your-exercise	NGO health information
	10	http://www.skimble.com/	App
	11	http://exercise.lbl.gov/	Government health information
	12	http://www.fitday.com/	Physical activity Online Health program
	13	https://itunes.apple.com/us/app/jefit-workout-fitness-bodybuilding/id449810000?mt=8	App
	14	http://www.bodybuilding.com/fun/printworklog.htm	Physical activity Online Health program
	15	http://www.sparkpeople.com/community/help_answer.asp?id=27	Physical activity Online Health program
	16	http://www.simplefitnesssolutions.com/articles/staying_on_track.htm	NGO health information
	17	http://lifehacker.com/5925850/google-tracks-20-turns-your-exercise-trips-into-eye+catching-data	Links to Online Health Program
	18	http://stopandbreathe.com/2011/03/19/strategies-to-get-your-exercise-program-back-on-track/	NGO health information
	19	http://dailyburn.com/	Physical activity Online Health program
	20	http://www.womenshealthmag.com/fitness/outdoor-workouts-1	Links to Online Health Program
	21	http://seanmullen.com/2012/07/13/tech-for-tracking-your-exercise-goals/	Links to Online Health Program
	22	http://www.myexerciseplan.com/	Physical activity Online Health program
	23	https://www.trackmytraining.co.uk/	Physical activity Online Health program
	24	http://go4life.nia.nih.gov/mygo4life/find-my-exercise-plans	Dead Link
	25	https://www.ifit.com/	Physical activity Online Health program
	26	http://www.shapefit.com/fittracker-exercise-journals.html	Physical activity Online Health program

	27	http://www.webmd.com/fitness-exercise/features/the-truth-about-heart-rate-and-exercise	NGO health information
	28	http://www.betterhealth.vic.gov.au/bhcv2/bhcarticles.nsf/pages/Exercise_intensity	Government health information
	29	http://www.dailymile.com/	App
	30	www.gymheroapp.com/	App

Free exercise program	1	http://www.workoutbox.com/workouts/	Physical activity Online Health program
	2	http://www.freetrainers.com/	Physical activity Online Health program
	3	http://www.whyiexercise.com/free-exercise-program.html	Physical activity Online Health program
	4	http://exercise.about.com/cs/weightloss/a/12weeks.htm	Links to Online Health Program
	5	http://www.muscleandstrength.com/workouts/main.html	Physical activity Online Health program
	6	http://www.noexcusesworkouts.com/free-exercise-programs-menu/	Links to Online Health Program
	7	http://www.acefitness.org/workouts/	Links to Online Health Program
	8	http://www.cairnsesplanade.com.au/Active%20Living%20-%20Fun%20Free%20Fitness	Other
	9	http://www.livestrong.com/free-fitness-programs/	Links to Online Health Program
	10	http://www.workoutz.com/	Links to Online Health Program
	11	http://www.myfreetrainer.com/	Links to Online Health Program
	12	http://www.marieclaire.com/health-fitness/news/power-up-workout-plan-week-1	Media Article
	13	http://www.makeuseof.com/tag/3-great-online-fitness-programs-shape/	Links to Online Health Program
	14	http://www.fitness.com/exercises/	Links to Online Health Program
	15	http://www.fitsync.com/	Links to Online Health Program
	16	http://www.bodyfitcheck.com.au/	Physical activity Online Health program
	17	http://www.noexcusesworkouts.com/	Physical activity Online Health program
	18	http://download.cnet.com/windows/health-and-fitness-software/	Links to Online Health Program
	19	http://www.myhomepersonaltrainer.com/	Physical activity Online Health program
	20	http://www.ironworkout.com/workout_programs/index.htm	Physical activity Online Health program
	21	http://www.fitday.com/fitness-articles/fitness/weight-loss/lose-15-pounds-in-1-month-free-sample-exercise-plan.html	Physical activity Online Health program
	22	http://fitbie.msn.com/slideshow/3-gym-free-workout-plans	Physical activity Online Health program
	23	http://www.gosnells.wa.gov.au/scripts/viewarticle.asp?NID=25641	Dead link
	24	http://scoobysworkshop.com/custom-workout-plans/	Physical activity Online Health program
	25	http://www.nowloss.com/free-weight-loss-workout-exercise-program.htm	Physical activity Online Health program
	26	http://au.lifestyle.yahoo.com/womens-health/fitness/speedy-workouts/	Physical activity Online Health program
	27	http://exercise.lifetips.com/cat/6003/exercise-plans/index.html	Links to Online Health Program

	28	http://www.personaltrainingprograms.com/	Physical activity Online Health program
	29	http://www.darebin.vic.gov.au/Page/Page.aspx?Page_Id=9515	Other
	30	http://www.oobafit.com/	Physical activity Online Health program

Health tracking online	1	http://www.preventionhealthtracker.com.au/	Physical activity Online Health program
	2	http://online.wsj.com/article/SB10001424052702304180804575188402688763416.html	Media Article
	3	http://www.healthehuman.com/	Physical activity Online Health program
	4	http://thecarrot.com/	Physical activity Online Health program
	5	http://greatist.com/health/complete-guide-to-tracking-health-and-fitness-online/	Media Article
	6	http://mashable.com/2011/09/23/the-complete-guide-to-tracking-health-fitness-online-infographic/	Media Article
	7	http://lifeboat.com/blog/2009/01/the-new-rise-of-online-health-tracking	Media Article
	8	https://myupmc.upmc.com/	Other
	9	http://www.healthunlocked.com/health-tracking/	Profile page
	10	http://lifehacker.com/5841439/five-best-fitness-tracking-web-sites-and-services	Links to Online Health Program
	11	http://www.thehealthcaregroup.com.au/pages/corporate/services/online-health-tracking-program.php	Profile page
	12	http://www.icpsr.umich.edu/icpsrweb/ICPSR/series/161	NGO health information
	13	http://ephtracking.cdc.gov/	Other
	14	http://www.governmenttechnology.co.uk/gt-news/item/2519-uks-first-online-health-tracker-launched	Media Article
	15	http://health.adelaide.edu.au/tracking/	Other
	16	http://health.adelaide.edu.au/tracking-applications/	Other
	17	http://www.heart.org/HEARTORG/Conditions/More/ToolsForYourHeartHealth/Keep-Track-of-Your-Heart-Health_UCM_318041_Article.jsp	Government health information
	18	http://www.prnewswire.com/news-releases/basis-health-tracker-and-web-service-reveals-new-way-to-make-lasting-improvements-to-fitness-and-sleep-181359501.html	Media Article
	19	http://mumbrella.com.au/prevention-launches-online-health-tracker-53633	Links to Online Health Program
	20	http://www.chartmyself.com/	NGO health information
	21	https://www.heart360.org/	Dead link
	22	http://www.forbes.com/forbes/2010/1206/investment-guide-medical-spending-health-care-tracking-vital-signs.html	Other
	23	http://tarasabo.blogspot.com/2012/01/tracking-your-health-and-fitness-online.html	NGO health information
	24	http://www.shapeup.com/news/article/shapeup-enhances-online-health-tracking-with-bodymedia-fit-armband-system-l	Links to Online Health Program

	25	http://gigaom.com/2012/10/30/note-to-startups-dont-forget-the-skinny-jeans-health-trackers/	NGO health information
	26	https://www.flshots.com/flshots/signin.csp	Other
	27	http://www.cnn.com/2009/HEALTH/expert.q.a/04/24/diet.tracking.sites.jampolis/index.html	Links to Online Health Program
	28	https://healthmanager.mayoclinic.com/help.aspx	Other
	29	http://www.hwia.com.au/pages/my-health-my-responsibility.php	Other
	30	http://www.kevinmd.com/blog/2012/04/gender-divide-health-tracking-online.html	Other

Health Tracking	1	http://health.adelaide.edu.au/tracking/	NGO health information
	2	http://www.preventionhealthtracker.com.au/	Physical activity Online Health program
	3	http://www.healthtrack.com.au/	Other
	4	http://www.bodyandsoul.com.au/health+healing/news+features/top+50+health+apps16209	NGO health information
	5	http://techland.time.com/2013/01/10/basis-health-tracking-watch-sports-serious-sensors-new-android-app/	Media Article
	6	http://www.chartmyself.com/	NGO health information
	7	http://fitbie.msn.com/fit-tracker	Online Health program
	8	https://hrtapp.com/	NGO health information
	9	http://www.mindmehealth.com/blog/tags/tag/health-tracking-software-1	Non PA Online Health Program
	10	http://venturebeat.com/2012/11/29/basis-science-reveals-its-health-tracking-wristwatch-and-fitness-web-service/	Links to Online Health Program
	11	http://www.kff.org/kaiserpolls/trackingpoll.cfm	Other
	12	http://www.icpsr.umich.edu/icpsrweb/HMCA/studies/34141	Research article
	13	http://www.forbes.com/sites/davidmaris/2013/01/12/health-tracking-gizmos-for-the-new-year/	Media Article
	14	https://myupmc.upmc.com/	Other
	15	http://healthmarketinnovations.org/program/community-health-information-tracking-system-chits	Profile page
	16	http://venturebeat.com/2013/01/06/bodymedia-launches-beefed-up-health-tracking-system-for-your-arm/	Media Article
	17	http://health.adelaide.edu.au/	Other
	18	http://www.utshealth.com/	Other
	19	http://thecarrot.com/	Physical activity Online Health program
	20	http://www.uq.edu.au/hishub/theme4	Other
	21	http://techcrunch.com/2012/11/29/basis-takes-on-jawbone-nike-finally-launches-its-impressive-199-health-tracking-band/	Media Article
	22	http://healthvermont.gov/tracking/	Government health information

	23	http://nwbc.com.au/resources/trackingforms.html	NGO health information
	24	http://www.ocregister.com/articles/health-367758-smarr-tracking.html	Media Article
	25	http://www.boston.com/lifestyle/health/gallery/wearable_trackers/	Media Article
	26	http://www.cnn.com/2012/09/21/health/quantified-self-data/index.html	Media Article
	27	http://msdn.microsoft.com/en-us/library/dd792678.aspx	Other
	28	http://msdn.microsoft.com/en-us/library/ee267758(v=bts.10).aspx	Other
	29	http://www.scientificamerican.com/citizen-science/project.cfm?id=health-tracking-network	Other
	30	http://www.medstudentsonline.com.au/f100/health-tracking-boh-24543/	Other

Free exercise websites	1	http://www.workoutz.com/	Physical activity Online Health program
	2	http://www.bodybuilding.com/exercises/	Physical activity Online Health program
	3	http://www.cairnsesplanade.com.au/Active%20Living%20-%20Fun%20Free%20Fitness	Other
	4	http://www.fitness.com/	Physical activity Online Health program
	5	http://www.livestrong.com/article/80108-exercise-videos-online/	Physical activity Online Health program
	6	http://fitness-websites.no1reviews.com/	Links to Online Health Program
	7	http://www.fitday.com/	Physical activity Online Health program
	8	http://www.freetrainers.com/	Physical activity Online Health program
	9	http://www.oobafit.com/	Physical activity Online Health program
	10	http://exercise.about.com/od/healthinjuries/a/internetinfo_2.htm	Links to Online Health Program
	11	http://www.fitsync.com/	Physical activity Online Health program
	12	http://www.myfitnesspal.com/topics/show/613477-free-online-exercise-websites	Physical activity Online Health program
	13	http://www.myfitnesspal.com/	Physical activity Online Health program
	14	http://www.exrx.net/	Physical activity Online Health program
	15	http://www.exercise4weightloss.com/	Physical activity Online Health program
	16	http://www.makeoverfitness.com/	Physical activity Online Health program
	17	http://www.acefitness.org/exerciselibrary/	Physical activity Online Health program
	18	http://www.darebin.vic.gov.au/Page/Page.aspx?Page_Id=9776	Other
	19	http://www.nycgovparks.org/befitnyc	Profile page
	20	http://www.nhs.uk/Livewell/fitness/Pages/Getfitwithoutgym.aspx	Government health information
	21	http://www.ilovefreesoftware.com/21/webware/free-websites-to-make-exercise-schedule.html	Links to Online Health Program
	22	http://www.myfitnesspal.com/topics/show/315302-free-workout-sites	Physical activity Online Health program
	23	http://www.physicalfitnet.com/	Physical activity Online Health program
	24	http://www.bodyrock.tv/	Physical activity Online Health program

	25	http://www.ibodyfit.com/	Dead Link
	26	http://www.thesaladgirl.com/best-workout-videos-free-exercise-routines/	Links to Online Health Program
	27	http://www.youtube.com/playlist?list=PLE4C5C45435CD7C95	Physical activity Online Health program
	28	http://shape.workoutmusic.com/	Other
	29	http://www.fitness-singles.com/	Other
	30	http://www.parentsconnect.com/parents/your-life/exercise-for-parents/customized-workout-routines-websites.html	Links to Online Health Program

Physical activity recording	1	http://www.walkgeorgia.org/index.cfm?public=UsingOnlineTools	Physical activity Online Health program
	2	http://www.cdc.gov/physicalactivity/data/surveillance.html	Government health information
	3	http://www.healthykids.nsw.gov.au/teachers-childcare/physical-activity-primary-school.aspx	Government health information
	4	http://www.nature.com/ijo/journal/v29/n3/full/0802882a.html	Research article
	5	http://www.ncbi.nlm.nih.gov/pubmed/15672111	Research article
	6	http://www.dhs.wisconsin.gov/health/physicalactivity/ToolCalcs.htm	Government health information
	7	http://sydney.edu.au/medicine/public-health/cpah/pdfs/2007_pa_measurement_farrell.pdf	Research article
	8	http://www.csa.com/factsheets/pei-set-c.php	Other
	9	http://cbrcc.curtin.edu.au/reports_journal_articles/ajpm%2041%2023-28.pdf	Research article
	10	http://www.atrf11.unisa.edu.au/Assets/Papers/ATRF11_0164_final.pdf	Government health information
	11	http://www.beactive.wa.gov.au/index.php?id=1395	Research article
	12	http://tde.sagepub.com/content/32/1/69.abstract	Research article
	13	http://www.ucsdeparc.org/index.php?option=com_content&view=article&id=94&Itemid=82	Other
	14	http://www.plosone.org/article/info%3Adoi%2F10.1371%2Fjournal.pone.0042202	Research article
	15	http://www.healthpromotion.com.au/Documents/CIM/Workplace_Challenge.pdf	Profile page
	16	http://www.scsepf.org/doc/210606/JESF0401-06.pdf	Research article
	17	http://www.ncbi.nlm.nih.gov/pubmed/19330930	Research article
	18	http://digitalcommons.library.umaine.edu/cgi/viewcontent.cgi?article=1095&context=etd	Research article
	19	http://www.10000steps.org.au/pdfs/conversion.pdf	Physical activity Online Health program
	20	http://www.health.qld.gov.au/ph/documents/hpu/healthykidsqld2006d.pdf	Government health information
	21	http://thepegeek.com/2010/03/31/the-physical-activity-log/	Physical activity Online Health program
	22	http://sma.org.au/2010/11/iphone-app-to-increase-physical-activity/	NGO health information
	23	http://www.tandfonline.com/doi/abs/10.1080/00071668508416845	Other

	24	http://www.health.gov.au/internet/main/publishing.nsf/content/66596E8FC68FD1A3CA2574D50027DB86/$File/childrens-nut-phys-survey.pdf	Government health information
	25	http://www.medicaljournals.se/jrm/content/download.php?doi=10.2340/16501977-0013	Research article
	26	http://www.archbronconeumol.org/	Dead link
	27	http://www.ncbi.nlm.nih.gov/pubmed/16898273	Research article
	28	http://informahealthcare.com/doi/pdf/10.3109/15412555.2012.708066	Dead link
	29	http://productzone.thoracicsurgerynews.com/term/8894/software-physiologic-recording-physical-activity	Other
	30	http://www.cqu.edu.au/research/research-organisations/institutes/health-and-social-sciences/centres2/centre-for-physical-activity-studies/research-outcomes/publications	Other

Exercise recording	1	http://www.shapefit.com/exercise-journals.html	Research article
	2	https://sites.google.com/a/worksmartlabs.com/help-center/cardiotrainer/recording-a-workout	Other
	3	http://www.weightlossresources.co.uk/exercise.htm	Physical activity Online Health program
	4	http://www.hws.edu/studentlife/counseling_relax.aspx	Other
	5	http://www.myfitnesspal.com/topics/show/453031-recording-exercise	Physical activity Online Health program
	6	http://www.fatsecret.com/Community.aspx?pa=fp&t=49618	Physical activity Online Health program
	7	http://www.zachpoff.com/teaching/projects-in-sound-art/recording-101/	Other
	8	http://www.copilotmanual.com/index.php?option=com_content&view=article&id=137&Itemid=270	Other
	9	http://www.polar.fi/e_manuals/Move/Polar_Move_user_manual_English/ch02.html	Other
	10	http://forums.watchuseek.com/f233/ambit-stopped-recording-74-minutes-mid-exercise-746966.html	Other
	11	http://www.androidtapp.com/jefit-workout-fitness-gymlog/jefit-exercise-recording/	Links to Online Health Program
	12	http://www.ncbi.nlm.nih.gov/pmc/articles/PMC483966/	Research article
	13	http://www.ncbi.nlm.nih.gov/pubmed/12964253	Research article
	14	http://www.ncbi.nlm.nih.gov/pubmed/17281036	Research article
	15	http://acronyms.thefreedictionary.com/Over-The-Horizon+Targeting+Exercise+Recording+Project	Other
	16	http://dombowerexercise.blogspot.com/2012/12/video-recording-your-workouts-benefits.html	Other
	17	http://www.writeenough.org.uk/recording_skills.htm	Other
	18	http://tei.oucs.ox.ac.uk/Talks/2009-07-oxford/exercise-08-Names_People_and_Places.pdf	Other
	19	http://www.weightware.com/exerciseeditor.htm	Physical activity Online Health program

	20	http://community.learnandmaster.com/index.php?/topic/24316-4-note-solo-exercise/	Other
	21	http://community.learnandmaster.com/index.php?/topic/20851-session-10-finger-style-beginner-exercise-recording/	Other
	22	http://iworkwell.ucol.ac.nz/	Business level online health program
	23	http://www.ncbi.nlm.nih.gov/pubmed/1006140	Research article
	24	http://contextualpsychology.org/recording_of_mind_train_defusion_exercise	Research article
	25	http://forum.bodybuilding.com/showthread.php?t=141753541&page=1	Physical activity Online Health program
	26	http://www.physio-pedia.com/File:Strengthening_exercise_recording_sheet.pdf	Other
	27	http://www.somaticmovementcenter.com/services/somaticexercises/	Other
	28	http://acapella.harmony-central.com/showthread.php?2948438-Recording-guitar-for-use-in-a-random-exercise-generator	Dead link
	29	http://www.facebook.com/pages/Jogingphysical-exercise-recording-muzik/515349138482489	Other
	30	http://www.comsys.rwth-aachen.de/teaching/ss-12/mit-lecture-and-exercise-video-recording/	Other

Walking tracker	1	http://www.mapmywalk.com/	Physical activity Online Health program
	2	http://www.geodistance.com/	Other
	3	http://walking.about.com/cs/measure/a/blhowtrack.htm	Links to Online Health Program
	4	https://itunes.apple.com/au/app/mapmywalk-gps-walking/id307861492?mt=8	App
	5	https://itunes.apple.com/us/app/walk-watch-gps-walking-computer/id384877736?mt=8	App
	6	http://www.justwalk.com/	Physical activity Online Health program
	7	http://startwalkingnow.org/mystart_tracker.jsp	Government health information
	8	http://walkertracker.com/	Physical activity Online Health program
	9	http://www.manlyaustralia.com.au/information/what_to_do/walksViews.asp	Other
	10	https://play.google.com/store/apps/details?id=com.mapmywalk.android2&hl=en	App
	11	http://www.androidcentral.com/google-slips-walking-and-cycling-tracking-card-google-now	Media Article
	12	http://www.sparkpeople.com/myspark/team_messageboard_thread.asp?board=19441x629x42879435	Physical activity Online Health program
	13	http://www.nbcnews.com/technology/futureoftech/phone-tracking-tech-reward-walking-biking-120038	Media Article
	14	http://www.popsci.com/technology/article/2011-10/gps-enabled-walking-shoes-help-caregivers-track-alzheimer%E2%80%99s-patients	Dead link

	15	http://www.walkingwithattitude.com/	Physical activity Online Health program
	16	http://www.mapmytracks.com/	App
	17	https://itunes.apple.com/us/app/walk-tracker-gps-fitness-tracker/id453030204?mt=8	App
	18	http://www.findanexpert.unimelb.edu.au/display/publication120801	Profile page
	19	http://www.petchecktechnology.com/	Other
	20	http://stepsout.com/	Business level online health program
	21	http://www.newzealand.com/int/walking-and-hiking/	Other
	22	http://www.ifp.illinois.edu/~hning2/papers/IVC04_ning%26tan.pdf	Research article
	23	http://short-walks.com.au/	Other
	24	http://ieeexplore.ieee.org/xpl/articleDetails.jsp?reload=true&arnumber=4938978	Research article
	25	http://ieeexplore.ieee.org/xpl/articleDetails.jsp?reload=true&arnumber=647403	Research article
	26	http://www.walkbiketoschool.org/keep-going/ongoing-activities/mileage-tracking	Physical activity Online Health program
	27	http://www.tripadvisor.com.au/ShowUserReviews-g319722-d1067198-r35809607-Nkuringo_Gorilla_Camp-Bwindi_Impenetrable_National_Park_Western_Region.html	Other
	28	http://walking.about.com/od/pedometerprogram/Pedometer_Walking_and_Step_Tracker_Programs.htm	Links to Online Health Program
	29	http://www.ywampublishing.com/p-258-tracking-your-walkbrthe-young-persons-prayer-diary.aspx	Other
	30	http://www.apartmenttherapy.com/let-tech-help-you-with-dog-walking-173043	Other

Fitness tracking	1	http://lifehacker.com/5841439/five-best-fitness-tracking-web-sites-and-services	Links to Online Health Program
	2	http://eftm.com.au/2013/01/ibitz-fitness-tracking-for-the-family-getting-the-kids-moving-9496	Media Article
	3	http://www.gymtechnik.com/	Physical activity Online Health program
	4	https://itunes.apple.com/au/app/jefit-workout-fitness-bodybuilding/id449810000?mt=8	App
	5	http://www.mapmyfitness.com/	Physical activity Online Health program
	6	http://dailyburn.com/	Online Health program
	7	http://au.ibtimes.com/articles/215820/20110919/best-of-the-best-top-five-fitness-tracking-web-sites.htm	Links to Online Health Program
	8	http://www.onlinefitnesslog.com/	Physical activity Online Health program
	9	http://www.lifehacker.com.au/2011/09/five-best-fitness-tracking-web-sites-and-services/	Links to Online Health Program
	10	https://www.bicyclenetwork.com.au/forums/viewtopic.php?f=14&t=21490	Other

	11	http://www.dakotafit.com/	Physical activity Online Health program
	12	http://www.myfitnesspal.com/	Physical activity Online Health program
	13	http://www.skimble.com/	App
	14	http://www.gymheroapp.com/	App
	15	http://support.xbox.com/games/dance-central/dance-central-3-fitness-tracking	Other
	16	http://www.lifespanfitness.com/lifespanfitnessclub.html	Physical activity Online Health program
	17	http://www.rubitrack.com/	App
	18	http://www.webmd.com/diet/food-fitness-planner/default.htm	Physical activity Online Health program
	19	http://gizmodo.com/fitness-tracking/	Media Article
	20	http://www.macrumors.com/2013/01/07/ces-2013-wearable-health-and-fitness-tracker-roundup/	Links to Online Health Program
	21	http://www.electricfoxy.com/2013/01/a-new-fitness-tracking-device/	Media Article
	22	http://fittracker.shapefit.com/	Physical activity Online Health program
	23	http://www.gymtechnik.com/blackberry/	App
	24	http://www.bodyplan.com/	Physical activity Online Health program
	25	http://now.msn.com/fitness-tracking-apps-raise-privacy-concerns	Media Article
	26	http://discussion.evernote.com/topic/28720-an-app-for-fitness-tracking-with-evernote/	App
	27	http://blog.visual.ly/visualizing-fitness-data/	Links to Online Health Program
	28	http://sites.google.com/site/healthnewsresources/quizzes-calculators-self-assessments-decision-guides/health-and-fitness-tracking-apps	Links to Online Health Program
	29	http://www.mensfitness.com/training/best-fitness-and-nutrition-apps-for-iphone	Media Article
	30	http://dailytekk.com/2012/06/11/fitness-tracking-devices/	Links to Online Health Program

Free exercise tracking	1	http://www.fitday.com/	Physical activity Online Health program
	2	http://www.onlinefitnesslog.com/	Physical activity Online Health program
	3	http://www.myfitnesspal.com/	Physical activity Online Health program
	4	https://itunes.apple.com/us/app/dailyburn-tracker/id378928008?mt=8	Links to Online Health Program
	5	http://www.livestrong.com/article/25929-workout-plan-creators/	Links to Online Health Program
	6	http://download.cnet.com/MedHelp-s-Exercise-Tracker/3000-2056_4-10909577.htm l	Links to Online Health Program
	7	http://www.builtlean.com/2012/01/27/workout-log-template/	Physical activity Online Health program
	8	http://lifehacker.com/5257812/six-best-exercise-planning-and-tracking-tools	Links to Online Health Program
	9	http://dailyburn.com/	Physical activity Online Health program
	10	http://www.fitsync.com/	Physical activity Online Health program

	11	http://bodyspace.bodybuilding.com/workouts/	Physical activity Online Health program
	12	http://www.bodybuilding.com/fun/printworklog.htm	Physical activity Online Health program
	13	http://www.fasttracktofatloss.com/	Links to Online Health Program
	14	http://www.examiner.com/article/usda-free-diet-and-exercise-tracking-tool-hits-one-million-user-mark	Links to Online Health Program
	15	http://www.freefitnesstracker.com/	Physical activity Online Health program
	16	http://greatist.com/health/best-health-and-fitness-apps/	Links to Online Health Program
	17	https://itunes.apple.com/us/app/exercise-tracker/id293603711?mt=8	App
	18	http://www.berryreview.com/2008/08/12/gym-technik-free-workout-tracking-on-your-blackberry/	Links to Online Health Program
	19	http://citeseerx.ist.psu.edu/viewdoc/download?doi=10.1.1.87.6500&rep=rep1&type=pdf	Research article
	20	https://play.google.com/store/apps/details?id=je.fit&hl=en	App
	21	http://voices.yahoo.com/top-5-free-iphone-diet-exercise-apps-6392942.html	Links to Online Health Program
	22	http://www.healthehuman.com/features/exercise-workout-tracker	Physical activity Online Health program
	23	http://fittracker.shapefit.com/	Online Health program
	24	http://www.phonicslessons.co.uk/trackingactivities.html	Other
	25	http://www.fitclick.com/	Physical activity Online Health program
	26	http://mashable.com/2009/01/03/free-iphone-apps-to-lose-weight/	Links to Online Health Program
	27	https://itunes.apple.com/en/app/body-fitness-free-ultimate/id343227312?mt=8	App
	28	http://www.lifehacker.com.au/2009/05/five-best-exercise-planning-and-tracking-tools/	Links to Online Health Program
	29	http://www.gymheroapp.com/	App
	30	http://www.warriorxfit.com/	Physical activity Online Health program

Exercise software	1	http://www.exercisesoftware.com/	Online Health program
	2	http://fitness-software-review.toptenreviews.com/	Links to Online Health Program
	3	http://www.proconditioning.com.au/	Commercial software
	4	http://www.physiotools.com/	Commercial software
	5	http://www.visualcoaching.com/	Profile page
	6	http://www.mavenlive.com/	Profile page
	7	http://www.physiotec.net.au/	Commercial software
	8	http://www.exrx.net/Store/Lists/Software.html	Profile page
	9	http://www.bioexsystems.com/product-line-exercise-software.aspx	Commercial software
	10	http://www.vhikits.com/	Profile page
	11	http://www.bioexsystems.com/	Commercial software

	12	http://www.bioexsystems.com/exercise-pro.aspx	Commercial software
	13	http://physicaltherapyweb.com/exercise_prescription_software.php	Commercial software
	14	http://www.fitnessbliss.com/	App
	15	http://en.wikipedia.org/wiki/List_of_exercise_prescription_software	Other
	16	http://www.edu-technology.com/exercisepro.html	Profile page
	17	http://www.simpleset.net/	Commercial software
	18	http://www.therehablab.com/	Commercial software
	19	http://www.proconditioning.com.au/index.php?option=com_content&view=article&id=76&Itemid=79	Commercial software
	20	http://www.accesshealth.com.au/exercise-products/exercise-software-programs/	Other
	21	http://www.capterra.com/physical-therapy-software	Links to Online Health Program
	22	http://guitar-and-bass-software.com/eng/exercise-list.html	Profile page
	23	http://training.strengthengine.com/	Commercial software
	24	http://www.biggly.com/	Commercial software
	25	http://www.dailyfitnesscenter.com/	Physical activity Online Health program
	26	http://www.dakotafit.com/	Physical activity Online Health program
	27	http://www.exercise-explorer.com/	Profile page
	28	http://www.us-cert.gov/reading_room/infosheet_Cyber%20Exercises.pdf	Government health information
	29	http://www.mavenlive.com/features	Profile page
	30	http://www.physx.com/	Profile page

Weight tracker	1	https://itunes.apple.com/au/app/weight-tracker/id284756469?mt=8	App
	2	http://www.3fatchicks.com/diet/weight-tracker/	Links to Online Health Program
	3	http://www.weighttracker.info/	Non PA Online Health Program
	4	http://www.fitwatch.com/fatpounders/	Physical activity Online Health program
	5	http://www.healthyweightforum.org/eng/weight/	Non PA Online Health Program
	6	http://www.tickerfactory.com/	App
	7	http://www.skinnyr.com/	Non PA Online Health Program
	8	http://download.cnet.com/My-Weight-Tracker/3000-2056_4-10909584.html	Links to Online Health Program
	9	https://play.google.com/store/apps/details?id=com.michaelfester.weighty&hl=en	App
	10	http://www.weight-tracker.buddyslim.com/	Non PA Online Health Program
	11	http://www.google.com.au/ig/directory?type=gadgets&url=www.google.com/ig/modules/google15.xml	Other
	12	http://www.tactiosoft.com/en/products/targetweight	App

	13	http://www.fitday.com/	Physical activity Online Health program
	14	https://play.google.com/store/apps/details?id=com.Weight_Tracker&hl=en	App
	15	http://www.mindmehealth.com/features/weight-tracker	Non PA Online Health Program
	16	http://www.windowsphone.com/en-us/store/app/weight-tracker/e6b400bf-911c-e011-9264-00237de2db9e	App
	17	https://itunes.apple.com/au/app/target-weight-for-adults-personal/id338889966?mt=8	App
	18	http://googlemodules.com/module/616/	Non PA Online Health Program
	19	http://appworld.blackberry.com/webstore/content/5780/	App
	20	http://www.babycenter.com/pregnancy-weight-gain-estimator	Other
	21	https://launchpad.net/wtods	Other
	22	http://www.hillspet.co.uk/en-gb/weight-management/weight-tracker-dog.html	Other
	23	http://appshopper.com/healthcare-fitness/pregnancy-weight-tracker	App
	24	http://www.doctoroz.com/videos/green-coffee-weight-tracker	Media Article
	25	http://blackberry-applications.toptenreviews.com/health-and-fitness/weight-tracker-review.html	Links to Online Health Program
	26	http://office.microsoft.com/en-us/templates/measurements-weight-tracker-TC001168403.aspx	Physical activity Online Health program
	27	http://itunes.apple.com/au/app/weight-tracker/id536045029?mt=8	App
	28	http://www.gerber.com/AllStages/growth_and_development/pregnancy_weight_chart.aspx	Non PA Online Health Program
	29	http://www.alfitness.com.au/	Physical activity Online Health program
	30	http://fitbie.msn.com/fit-tracker	Physical activity Online Health program

Calorie exercise tracker	1	http://www.myfitnesspal.com/	Physical activity Online Health program
	2	http://www.wired.com/playbook/2012/08/fitness-trackers/	Links to Online Health Program
	3	http://www.webmd.com/diet/food-fitness-planner/default.htm	Physical activity Online Health program
	4	http://www.webmd.com/diet/healthtool-fitness-calorie-counter	Physical activity Online Health program
	5	http://www.livestrong.com/thedailyplate/	Physical activity Online Health program
	6	http://www.my-calorie-counter.com/	Non PA Online Health Program
	7	http://www.fitday.com/	Physical activity Online Health program
	8	https://itunes.apple.com/us/app/tap-track-calorie-counter/id307749752?mt=8	App
	9	http://www.nytimes.com/2012/01/05/technology/personaltech/devices-to-keep-track-of-calories-lost-or-gained.html?pagewanted=all	Media Article
	10	http://www.fitwatch.com/diary/activitydiary.html	Physical activity Online Health program
	11	http://www.mynetdiary.com/	Physical activity Online Health program

	12	http://www.healthstatus.com/calculate/cbc	Other
	13	http://caloriecount.about.com/	Non PA Online Health Program
	14	http://www.myfooddiary.com/	Physical activity Online Health program
	15	http://www.148apps.com/reviews/tap-track-calorie-weight-exercise-tracker/	App
	16	http://www.windowsphone.com/en-au/store/app/calorie-tracker/1229f984-2e99-e011-986b-78e7d1fa76f8	App
	17	https://itunes.apple.com/us/app/dailyburn-tracker/id378928008?mt=8	App
	18	http://mashable.com/2009/01/03/free-iphone-apps-to-lose-weight/	Links to Online Health Program
	19	http://www.macworld.com/product/79923/tap-track-calorie-tracker.html	App
	20	http://www.exercise4weightloss.com/calorie-intake-chart.html	Physical activity Online Health program
	21	http://iphone.appstorm.net/roundups/lifestyle-roundups/10-iphone-apps-for-counting-calories/	Media Article
	22	http://www.caloriesperhour.com/	Non PA Online Health Program
	23	http://calorie-tracker.thelongmores.me.uk/	Non PA Online Health Program
	24	https://chrome.google.com/webstore/detail/diet-diary/neckeibmjhibmgoigmffjlihekefmffd	App
	25	http://www.fitclick.com/calories_burned	Physical activity Online Health program
	26	http://www.fitclick.com/	Physical activity Online Health program
	27	http://ucanrow2.com/myfitnesspal-calorieexercise-tracker-gets-a-huge-upgrade/	Links to Online Health Program
	28	https://itunes.apple.com/us/app/tracknburn-calorie-diet-exercise/id385920654?mt=8	App
	29	http://calorieline.com/	Physical activity Online Health program
	30	http://www.primusweb.com/fitnesspartner/calculat.htm	Physical activity Online Health program

Calorie tracker	1	http://www.myfitnesspal.com/	Physical activity Online Health program
	2	http://www.my-calorie-counter.com/	Non PA Online Health Program
	3	https://itunes.apple.com/au/app/livestrong.com-calorie-tracker/id295305241?mt=8	App
	4	http://www.fitday.com/about	Physical activity Online Health program
	5	http://www.livestrong.com/thedailyplate/	NGO health information
	6	http://www.webmd.com/diet/food-fitness-planner/default.htm	Physical activity Online Health program
	7	http://www.preventionhealthtracker.com.au/	Physical activity Online Health program
	8	http://www.wired.com/playbook/2012/08/fitness-trackers/	Links to Online Health Program
	9	https://appworld.blackberry.com/webstore/content/7152/	App
	10	http://ask.metafilter.com/177457/Whats-the-current-bestinclass-calorietracking-website	Links to Online Health Program

	11	http://www.perfect-diet-tracker.com/	Non PA Online Health Program
	12	http://9gag.com/gag/4951128	Other
	13	http://www.calorieking.com.au/	Physical activity Online Health program
	14	http://abcnews.go.com/Health/story?id=5420269&page=1	Media Article
	15	http://www.macworld.com.au/app-guide/calorie-counter-diet-tracker-59091/	App
	16	http://www.cbc.ca/news/background/food/restaurant-tracker.html	Other
	17	https://itunes.apple.com/au/app/calorie-counter-diet-tracker/id341232718?mt=8	App
	18	http://store.ovi.com/content/178820	App
	19	http://blog.programmableweb.com/2012/07/06/be-a-calorie-tracker-hacker-with-mynetdiary/	Other
	20	http://www.eatright.org/WorkArea/linkit.aspx?LinkIdentifier=id&ItemID=6442467034&libID=6442467016	App
	21	https://itunes.apple.com/us/app/livestrong.com-calorie-tracker/id502317923?mt=8	App
	22	https://itunes.apple.com/au/app/australian-calorie-counter/id436104108?mt=8	App
	23	http://www.nytimes.com/2012/01/05/technology/personaltech/devices-to-keep-track-of-calories-lost-or-gained.html?pagewanted=all	Links to Online Health Program
	24	http://www.changemyplate.com/calorie-tracker/	Physical activity Online Health program
	25	http://www.findingoptimism.com/blog/apps/review-of-livestrong-calorie-tracker/	App
	26	http://www.pcmag.com/article2/02817239335900.asp	Dead Link
	27	http://www.shape.com/fitness/training-plans/best-free-apps-runners?page=6	Links to Online Health Program
	28	http://www.builtlean.com/2010/06/15/best-free-online-calorie-tracker-and-app/	Links to Online Health Program
	29	http://www.macworld.com/product/79923/tap-track-calorie-tracker.html	App
	30	http://www.alfitness.com.au/	Physical activity Online Health program

Free weight programs	1	http://tracker.dailyburn.com/workout_programs/55291-5-Day-Split-Free-Weights	Physical activity Online Health program
	2	http://tracker.dailyburn.com/workout_programs/34-All-Free-Weights	Physical activity Online Health program
	3	http://weighttraining.about.com/od/succeedingwithweights/a/strength_muscle.htm	Physical activity Online Health program
	4	http://www.trulyhuge.com/free_weights.htm	Physical activity Online Health program
	5	http://www.realsolutionsmag.com/weight-training/	Physical activity Online Health program
	6	http://www.freedieting.com/	Links to Online Health Program
	7	http://www.freetrainers.com/	Physical activity Online Health program

	8	http://www.sparkpeople.com/	Physical activity Online Health program
	9	http://www.fitclick.com/	Physical activity Online Health program
	10	http://www.nowloss.com/free-weight-loss-workout-exercise-program.htm	Physical activity Online Health program
	11	http://www.powerzonenutrition.com.au/main/index.php?option=com_content&view=article&id=86&Itemid=86	Physical activity Online Health program
	12	http://www.startyourdiet.com/	Physical activity Online Health program
	13	http://fittplan.com/free-weights-workout-program	Physical activity Online Health program
	14	http://en.wikipedia.org/wiki/Weight_training	NGO health information
	15	http://www.exercise4weightloss.com/free-weight-loss-programs.html	Physical activity Online Health program
	16	http://voices.yahoo.com/the-best-free-online-weight-loss-programs-5622216.html	Links to Online Health Program
	17	http://43weightlossexercise.webs.com/	Links to Online Health Program
	18	http://www.muscleandstrength.com/workouts/main.html	Physical activity Online Health program
	19	http://www.personaltrainingprograms.com/	Physical activity Online Health program
	20	http://hasfit.com/workouts/weight-loss/	Physical activity Online Health program
	21	http://www.freedieting.com/exercise_plans.htm	Physical activity Online Health program
	22	http://www.livestrong.com/free-weight-loss-programs/	Physical activity Online Health program
	23	http://www.isowhey.com.au/assets/pdf/002_EbookIsoWHey_Booklet.pdf	Other
	24	http://www.dailymotion.com/video/x8yoli_free-weight-lifting-programs-offers_news	Other
	25	http://www.dailymotion.com/video/x7ctvb_free-weight-loss-programs_news	Other
	26	http://idealbody4life.com/free-weight-loss-exercise-programs	Physical activity Online Health program
	27	http://www.menshealth.co.uk/lose-weight/	Physical activity Online Health program
	28	http://www.facebook.com/pages/Free-Weight-Loss-Tips-and-Weight-Training-Programs/152097804800590	Physical activity Online Health program
	29	http://www.sallysymonds.com.au/free-stuff/47-resources/free-resources/58-the-best-free-online-diet-and-exercise	Links to Online Health Program
	30	http://www.hmrprogram.com/index.cfm/Programs/ClinicPrograms/Decision-Free_Program	Non PA Online Health Program

Weight loss program	1	http://www.weightloss.com.au/weight-loss-programs.html	Physical activity Online Health program
	2	http://www.tonyferguson.com/	Non PA Online Health Program
	3	http://www.bodytrim.com.au/	Non PA Online Health Program
	4	http://weightlossprogramsaustralia.com/	Non PA Online Health Program
	5	http://www.productreview.com.au/c/diets-weight-loss-programs.html	Links to Online Health Program
	6	http://www.sureslim.com.au/	Non PA Online Health Program
	7	http://www.alfitness.com.au/	Physical activity Online Health program

	8	http://www.wesweight.com.au/weight-loss-program	Non PA Online Health Program
	9	http://www.jennycraig.com.au/weight-loss-programs	Non PA Online Health Program
	10	http://www.ultralite.com.au/	Non PA Online Health Program
	11	http://www.smh.com.au/national/health/lifetime-of-dieting-is-the-new-weight-loss-plan-20121229-2c0l2.html	Non PA Online Health Program
	12	http://biggestloserclub.com.au/	Physical activity Online Health program
	13	http://www.fatblaster.com.au/weight-loss-program/	Physical activity Online Health program
	14	http://www.fatblaster.com.au/downloadWeightLossBooklet.php	Non PA Online Health Program
	15	http://www.orberasystem.com.au/	Non PA Online Health Program
	16	http://www.bmiweightloss.com.au/	Physical activity Online Health program
	17	http://www.themainmeal.com.au/Red+meat+and+nutrition/Weight-loss-plan-young-women/	Non PA Online Health Program
	18	http://www.weightloss.com.au/diy-weight-loss-program.html	Physical activity Online Health program
	19	http://www.drtims.com.au/	Non PA Online Health Program
	20	http://www.bodybalancing.com.au/	Physical activity Online Health program
	21	http://changinghabits.com.au/21-day-healthy-weight-loss-program	Non PA Online Health Program
	22	http://www.bupa.com.au/health-and-wellness/programs-and-support/living-well-programs/weight-management-program	Physical activity Online Health program
	23	http://www.cohens.com.au/our_program/about_cohens_weight_loss_program.htm	Non PA Online Health Program
	24	http://www.weightwatchers.com.au/plan/apr/index.aspx	Physical activity Online Health program
	25	http://www.fitnessmagazine.com/weight-loss/plans/	Physical activity Online Health program
	26	https://www.treatyourselfwell.com.au/	Physical activity Online Health program
	27	http://www.wesweight.com.au/	Non PA Online Health Program
	28	http://www.eatingwell.com/nutrition_health/weight_loss_diet_plans/diet_meal_plans/7_day_diet_meal_plan_to_lose_weight_1200_calories	Non PA Online Health Program
	29	http://www.donnaandtora.com/programs/ultimate-weight-control-formula/	Non PA Online Health Program
	30	http://amcal.begoodtoyourself.com.au/amcal/our-program/	Physical activity Online Health program

Onilne exercise program	1	http://www.trainwithmeonline.com/	Physical activity Online Health program
	2	http://www.warriorxfit.com/	Physical activity Online Health program
	3	http://www.foxnews.com/health/2011/05/20/10-online-workout-programs/	Links to Online Health Program
	4	http://www.oobafit.com/	Physical activity Online Health program
	5	http://www.freetrainers.com/	Physical activity Online Health program
	6	http://www.askmen.com/top_10/fitness/top-10-online-workout-programs.html	Links to Online Health Program
	7	http://www.alfitness.com.au/	Physical activity Online Health program

	8	http://www.physiotools.com/	Commercial software
	9	http://www.sallysymonds.com.au/free-stuff/47-resources/free-resources/58-the-best-free-online-diet-and-exercise	Physical activity Online Health program
	10	http://www.idealbodiesonline.com/	Physical activity Online Health program
	11	http://www.workoutsforyou.com/	Physical activity Online Health program
	12	http://www.therehablab.com/	Commercial software
	13	http://www.proconditioning.com.au/	Commercial software
	14	http://www.proconditioning.com.au/index.php?option=com_content&view=article&id=76&Itemid=79	Commercial software
	15	http://physicaltherapyweb.com/exercise_prescription_software.php	Commercial software
	16	http://www.physiotec.ca/	Commercial software
	17	http://www.workouttrainer.com/	Physical activity Online Health program
	18	http://www.slimtree.com/	Physical activity Online Health program
	19	http://www.intrafitt.com/	Physical activity Online Health program
	20	http://www.workoutz.com/	Physical activity Online Health program
	21	http://www.makeuseof.com/tag/3-great-online-fitness-programs-shape/	Links to Online Health Program
	22	http://www.sportecoach.com.au/online-training-programs.html	Physical activity Online Health program
	23	http://www.sparkpeople.com/resource/videos.asp	Physical activity Online Health program
	24	http://www.myexerciseplan.com/	Physical activity Online Health program
	25	http://www.dailymail.co.uk/health/fitnessWorkOutArms.html	Physical activity Online Health program
	26	http://online-fitness-services-review.toptenreviews.com/	Links to Online Health Program
	27	http://www.hep2go.com/	Commercial software
	28	http://www.gymamerica.com/	Physical activity Online Health program
	29	http://www.myhomepersonaltrainer.com/home/index.htm	Physical activity Online Health program
	30	http://www.fitwatch.com/planner/workoutplanner.html	Physical activity Online Health program

Health tracking software	1	http://www.mindmehealth.com/blog/tags/tag/health-tracking-software-1	Life coaching
	2	http://download.cnet.com/Health-Tracker/3000-2129_4-73404.html	App
	3	http://howto.wired.com/wiki/Use_Tech_to_Track_Your_Health	Links to Online Health Program
	4	http://www.ilovefreesoftware.com/21/windows/free-health-tracking-software-my-daily-readings.html	Profile page
	5	http://www.facebook.com/benutriwise	Profile page
	6	http://www.omronhealthcare.com/service-and-support/connected-health/	Profile page
	7	http://mashable.com/2011/11/06/apps-health-productivity/	Links to Online Health Program
	8	http://www.healthtrack.com.au/	Profile page
	9	http://www.integritas.com/employee-health-software.html	Profile page

	10	http://ieeexplore.ieee.org/xpl/articleDetails.jsp?reload=true&arnumber=336779	Research article
	11	http://www.cdc.gov/nceh/tracking/pdfs/how_to_guide.pdf	Research article
	12	http://www.puresafety.com/public/products-services/occupational-health-manager-ohm	Profile page
	13	http://www.techsoftwaremarket.com/health-tracking-app/	Other
	14	https://play.google.com/store/apps/details?id=com.benoved.phr_lite	App
	15	http://readyset.axionhealth.com/	Other
	16	http://www.seratec.com.au/industry/local_council/council-health-inspection-tracking-software.htm	Other
	17	http://mobile.brothersoft.com/download/health-tracker.html	Other
	18	http://ieeexplore.ieee.org/xpls/abs_all.jsp?arnumber=336779	Other
	19	http://www.cbsnews.com/2100-500165_162-6417556.html	Links to Online Health Program
	20	http://www.recordsforliving.com/HealthFrame/Feature_ExpenseTracking.aspx	Other
	21	http://www.youtube.com/user/HowsMyPet/videos	Other
	22	http://branch.com/b/why-does-almost-all-health-tracking-software-hardware-suck?ref=just_started	Other
	23	http://www.forbes.com/forbes/2010/1206/investment-guide-medical-spending-health-care-tracking-vital-signs.html	Other
	24	http://www.fitstatsweb.com/	Commercial software
	25	http://www.tanita.com/en/tanita-health-ware/	Profile page
	26	http://pctsrajmedical.raj.nic.in/	Research article
	27	http://venturebeat.com/2012/11/29/basis-science-reveals-its-health-tracking-wristwatch-and-fitness-web-service/	Links to Online Health Program
	28	http://www.cdc.gov/nceh/tracking/	Government health information
	29	http://forums.anandtech.com/showthread.php?p=34328611	Other
	30	http://www.weightbydate.com/pro_overview_fitness.htm	Physical activity Online Health program

Environmental health tracking	1	http://www.cdc.gov/nceh/tracking/	Government health information
	2	http://ephtracking.cdc.gov/	Government health information
	3	http://www.public.health.wa.gov.au/2/1398/2/environmental_health_surveillance.pm	Government health information
	4	https://tracking.publichealth.maine.gov/	Government health information
	5	http://www.cehtp.org/	Government health information
	6	http://healthvermont.gov/tracking/	Government health information
	7	http://www.hpa.org.uk/ProductsServices/ChemicalsPoisons/EnvironmentalPublicHealthTracking/	Government health information
	8	http://www.jhsph.edu/research/centers-and-institutes/center-for-	Government health information

		excellence-in-environmental-health-tracking/index.html	
	9	http://ehtracking.berkeley.edu/	NGO health information
	10	http://www.health.state.mn.us/tracking/	Government health information
	11	http://www.facebook.com/CDCEPHTracking	Profile page
	12	http://a816-dohbesp.nyc.gov/IndicatorPublic/	Government health information
	13	http://www.health.ny.gov/statistics/environmental/public_health_tracking/	Government health information
	14	http://public.health.oregon.gov/HealthyEnvironments/EnvironmentalExposures/EnvironmentalPublicHealthTracking/	Research article
	15	http://www.portal.state.pa.us/portal/server.pt?open=514&objID=557488&mode=2	Government health information
	16	http://esriaustralia.com.au/u/lib/cms/esri-health_whitepaper.pdf	Research article
	17	http://www.cdc.gov/features/trackingnetwork/	Government health information
	18	http://www.naccho.org/topics/environmental/EPHT/index.cfm	Government health information
	19	http://www.ncbi.nlm.nih.gov/pmc/articles/PMC2653708/	Research article
	20	http://ideha.dhmh.maryland.gov/OEHFP/EH/tracking/SitePages/Home.aspx	Government health information
	21	http://www.naphsis.org/Pages/EnvironmentalPublicHealthTrackingOV.aspx	Government health information
	22	http://www.dh.sa.gov.au/pehs/publications/0812-EH-indicators-discussion-paper.pdf	Research article
	23	http://www.cddep.org/projects/cdc_environmental_public_health_tracking_network_evaluation	Government health information
	24	http://www.scdhec.gov/administration/epht/	Government health information
	25	http://matracking.ehs.state.ma.us/	Government health information
	26	http://www.coepht.dphe.state.co.us/	Government health information
	27	http://ephtracking.cdc.gov/showPopCharEnv.action	Government health information
	28	http://www.nbdpn.org/docs/TuesBreakout_330PM_EPHT_Strosnider_WEB.pdf	Research article
	29	http://www.ncbi.nlm.nih.gov/pmc/articles/PMC1247569/	Research article
	30	http://www.ct.gov/dph/cwp/view.asp?a=3140&q=386922&dphNav_GID=1826	Government health information

Online health tracking	1	http://online.wsj.com/article/SB10001424052702304180804575188402688763416.html	Links to Online Health Program
	2	http://greatist.com/health/complete-guide-to-tracking-health-and-fitness-online/	Links to Online Health Program
	3	http://mashable.com/2011/09/23/the-complete-guide-to-tracking-health-fitness-online-infographic/	Links to Online Health Program
	4	http://www.preventionhealthtracker.com.au/	Physical activity Online Health program
	5	http://www.healthehuman.com/	Physical activity Online Health program

	6	https://www.heart360.org/	Physical activity Online Health program
	7	http://www.msnbc.msn.com/id/16526412/ns/health-heart_health/t/online-tracker-monitors-calories-exercise/	Media Article
	8	http://www.thehealthcaregroup.com.au/pages/corporate/services/online-health-tracking-program.php	Physical activity Online Health program
	9	https://myupmc.upmc.com/	Other
	10	http://www.governmenttechnology.co.uk/gt-news/item/2519-uks-first-online-health-tracker-launched	Other
	11	http://www.shapeup.com/news/article/shapeup-enhances-online-health-tracking-with-bodymedia-fit-armband-system-l	Physical activity Online Health program
	12	http://lifeboat.com/blog/2009/01/the-new-rise-of-online-health-tracking	Links to Online Health Program
	13	http://www.hwia.com.au/pages/my-health-my-responsibility.php	Links to Online Health Program
	14	http://www.pewinternet.org/Reports/2011/Social-Life-of-Health-Info.aspx	Research article
	15	http://www.prnewswire.com/news-releases/basis-health-tracker-and-web-service-reveals-new-way-to-make-lasting-improvements-to-fitness-and-sleep-181359501.html	Links to Online Health Program
	16	http://tarasabo.blogspot.com/2012/01/tracking-your-health-and-fitness-online.html	Other
	17	http://www.prevention.com/	Physical activity Online Health program
	18	https://healthmanager.mayoclinic.com/help.aspx	Other
	19	http://www.chartmyself.com/	Physical activity Online Health program
	20	http://www.zeroonezero.com/design/website-copywriting/toni/online-health-tracking/	Other
	21	http://pctsrajmedical.raj.nic.in/	Other
	22	http://www.wellsource.com/health-activity-tracker.html	Physical activity Online Health program
	23	http://powerinfographics.wordpress.com/2013/01/06/guide-for-tracking-your-health-and-fitness-online/	Dead Link
	24	http://www.heart.org/HEARTORG/Conditions/More/ToolsForYourHeartHealth/Keep-Track-of-Your-Heart-Health_UCM_318041_Article.jsp	Physical activity Online Health program
	25	http://www.icpsr.umich.edu/icpsrweb/ICPSR/series/161	Research article
	26	http://www.medhelp.org/land/health-trackers	Links to Online Health Program
	27	http://www.pewinternet.org/Shared-Content/Data-Sets/2010/September-2010--Health.aspx	Links to Online Health Program
	28	http://www.cmbinfo.com/cmb-cms/wp-content/uploads/2012/11/Match.com_CaseStudy.pdf	Other
	29	http://health.adelaide.edu.au/tracking/	Other
	30	http://venturebeat.com/2012/11/29/basis-science-reveals-its-health-tracking-wristwatch-and-fitness-web-service/	Links to Online Health Program

GPS walking tracker	1	https://itunes.apple.com/au/app/walk-tracker-gps-fitness-tracker/id453030204?mt=8	App

	2	https://itunes.apple.com/au/app/walk-watch-gps-walking-computer/id384877736?mt=8	App
	3	http://walking.about.com/od/pedometer1/tp/gadgets.htm	Other
	4	http://www.walkgps.com/GPS%20Navigation.htm	Other
	5	https://play.google.com/store/apps/details?id=com.charsoftware.android.gps.walkandruntracker&hl=en	App
	6	https://play.google.com/store/apps/details?id=com.fitnesskeeper.runkeeper.pro&hl=en	App
	7	http://www.garmin.com/uk/company/about_us/	Profile page
	8	http://gpstracklog.com/buyers-guides/gps-for-hiking	Other
	9	http://www.digitalworldz.co.uk/260168-android-gps-tracker-walking.html	Other
	10	http://uk.answers.yahoo.com/question/index?qid=20110405135919AAwpfAj	Links to Online Health Program
	11	http://uk.answers.yahoo.com/question/index?qid=20100131144718AAKVAaK	Other
	12	http://mobile.brothersoft.com/gps-walk-and-run-tracker-74503.html	App
	13	https://getsatisfaction.com/swingbyswing/topics/gps_walking_tracker_cool	App
	14	http://androidforums.com/htc-hero/37495-looking-accurate-walking-gps-tracker.html	App
	15	http://gps-tracker-review.toptenreviews.com/ecco-personal-gps-locator-review.html	Other
	16	http://pathtracks.com/	App
	17	https://itunes.apple.com/au/app/mapmywalk-gps-walking/id307861492?mt=8	App
	18	http://trails.lamouroux.de/	App
	19	http://handheld.softpedia.com/get/GPS/GPS-Sport-Tracker-92379.shtml	App
	20	http://www.outdoorsafetygps.com/	Other
	21	http://news.idealo.co.uk/news/2756/gps-tracking-watches.html	Other
	22	http://home.trainingpeaks.com/mobile-apps/iphone-trainingpeaks-gps.aspx	App
	23	http://appshopper.com/healthcare-fitness/walkwatch-gps-walking-computer-for-tracking-mapping-and-fitness	App
	24	http://www.optimaltracking.com/en/gps-randonnees-plein-air	Other
	25	http://gps-tracker-review.toptenreviews.com/	Other
	26	http://www.alibaba.com/showroom/walking-tracker.html	Other
	27	https://itunes.apple.com/en/app/walktastic-gps-nordic-walking/id380335165?mt=8	App
	28	http://www.freewarepocketpc.net/ppc-download-gps-sport-tracker.html	App
	29	http://www.petchecktechnology.com/	Other
	30	http://www.aliexpress.com/wholesale/wholesale-walking-tracker.html	Other

Walking Tracking App	1	https://itunes.apple.com/us/app/walk-tracker-pro/id423400390?mt=8	App
	2	https://itunes.apple.com/us/app/mapmywalk-gps-walking/id307861492?mt=8	App
	3	https://play.google.com/store/apps/details?id=com.charsoftware.android.gps.walkandruntracker&hl=en	App
	4	https://play.google.com/store/apps/details?id=com.highwaynorth.jogtracker&hl=en	App
	5	https://play.google.com/store/apps/details?id=com.endomondo.android&hl=en	App
	6	http://walking.about.com/od/maps/tp/Best-Walking-App-Readers-Choice-Awards-2012.htm	App
	7	http://www.abvio.com/walkmeter/	App
	8	http://www.mapmywalk.com/	App
	9	http://www.mapmywalk.com/imapmy/iphone/	App
	10	http://support.trainingpeaks.com/trainingpeaks-apps/trainingpeaks-gps-apps/walktracker-pro.aspx	App
	11	http://apple.stackexchange.com/questions/51883/whats-a-good-sports-tracker-app-for-iphone	App
	12	http://www.appszoom.com/android_applications/gps+walk+and+run+tracker	App
	13	http://www.jogtracker.com/	App
	14	http://appshopper.com/mac/healthcare-fitness/walk-tracker	App
	15	http://mashable.com/2009/01/03/free-iphone-apps-to-lose-weight/	App
	16	http://www.geodistance.com/	Other
	17	https://itunes.apple.com/us/app/endomondo-sports-tracker-gps/id333210180?mt=8	App
	18	http://www.everythingicafe.com/forum/threads/tracking-your-running-walking-cycling-hiking-etc.31627/	App
	19	http://www.iphoneincanada.ca/app-store/starbucks-app-pick-of-the-week-walk-tracker-pro/	App
	20	http://www.bodyandsoul.com.au/health+healing/news+features/top+50+health+apps16209	Other
	21	http://www.topiphoneapplication.com/walking-distance-tracking-app/	App
	22	http://startwalkingnow.org/mystart_tracker.jsp	Physical activity Online Health program
	23	http://howto.cnet.com/8301-11310_39-57349556-285/five-fitness-tracking-apps-for-android/	App
	24	http://www.outsideonline.com/fitness/Hit-the-Road-app.html	App
	25	http://pathtracks.com/	App
	26	http://www.facebook.com/walkertracker	Profile page
	27	https://itunes.apple.com/us/app/walk-tracker-gps-fitness-	App

		tracker/id453030204?mt=8	
	28	http://tracker.dailyburn.com/workout_programs/73233	App
	29	http://www.everytrail.com/iphone.php	App
	30	http://slickdeals.net/f/4789612-Starbucks-App-Codes-Walk-Tracker-Pro-Cat-In-The-Hat	Other

Walking distance tracker	1	http://www.geodistance.com/	Other
	2	http://www.gmap-pedometer.com/	Other
	3	http://www.mapmywalk.com/	Physical activity Online Health program
	4	https://itunes.apple.com/us/app/mapmywalk-gps-walking/id307861492?mt=8	App
	5	http://www.jogtracker.com/	App
	6	http://walking.about.com/library/walk/blgooglemap1.htm	App
	7	http://www.walkjogrun.net/	Physical activity Online Health program
	8	http://www.abvio.com/walkmeter/	App
	9	https://play.google.com/store/apps/details?id=com.endomondo.android&hl=en	App
	10	http://www.topiphoneapplication.com/walking-distance-tracking-app/	App
	11	https://play.google.com/store/apps/details?id=com.fitnesskeeper.runkeeper.pro&hl=en	App
	12	http://startwalkingnow.org/mystart_tracker.jsp	Physical activity Online Health program
	13	http://mashable.com/2010/02/23/iphone-apps-runners/	Links to Online Health Program
	14	http://tracker.dailyburn.com/workout_programs/73233	Physical activity Online Health program
	15	http://downloads.yahoo.com/software/mobile-iphone-walking-distance-meter-s550713	App
	16	http://walking.about.com/cs/measure/a/blhowtrack.htm	Links to Online Health Program
	17	http://www.droidforums.net/forum/droid-incredible-general-discussions/42544-there-app-tracks-miles-i-walk-run.html	Links to Online Health Program
	18	http://www.rei.com/learn/expert-advice/fitness-monitors.html	Other
	19	http://www.everythingicafe.com/forum/threads/tracking-your-running-walking-cycling-hiking-etc.31627/	Links to Online Health Program
	20	http://www.amazon.com/GOsmart-Pace-Distance-Tracker-Pedometers/dp/B0055QHM14	Other
	21	http://tracker.dailyburn.com/forums/fitness_and_exercise/topics/calculate_walking_distance_with_google_maps	Links to Online Health Program
	22	http://www.brighthub.com/mobile/blackberry-platform/articles/96496.aspx	Links to Online Health Program
	23	http://androidforums.com/htc-hero/37495-looking-accurate-walking-gps-tracker.html	Links to Online Health Program
	24	http://uk.ask.com/beauty/Walking-Distance-Tracker	Other

	25	http://www.androidcentral.com/google-slips-walking-and-cycling-tracking-card-google-now	App
	26	http://walking.about.com/od/maps/tp/Best-Walking-App-Readers-Choice-Awards-2012.htm	App
	27	http://www.androidguys.com/2012/09/07/five-for-friday-android-apps-for-tracking-a-run/	App
	28	http://www.topappcharts.com/433415717/app-details-walking-distance-meter.php	App
	29	http://www.livestrong.com/article/241650-calculate-walking-calories-by-distance/	NGO health information
	30	http://www.vertex42.com/ExcelTemplates/running-log.html	Other
